# Supplementary material for: The application of the propensity score matching method in stock prediction among stocks within the same industry
Source: PeerJ Comput Sci. 2024 Jan 30;10:e1819. doi: 10.7717/peerj-cs.1819 (PMC10909155; doi:10.7717/peerj-cs.1819)
Supplement: Supplemental Information 1 [file peerj-cs-10-1819-s001.pdf]

Appendix 1

Biopharmaceutical Sub-Industry Stock Price Forecast Analysis

In the biopharmaceutical sub-industry, the top five companies by market capitalization are Beigene, Ltd (stock code: 688235.sh), Shanghai Junshi Biosciences Co., Ltd (stock code: 688180.sh, hereafter referred to as "Junshi"), Cansino Biologics Inc (stock code: 688185.sh), Beijing Tiantan Biological Products Corporation Limited (stock code: 600161.sh, hereafter referred to as "Tiantan"), and Nanjing King-Friend Biochemical Pharmaceutical Co., Ltd (stock code: 603707.sh, hereafter referred to as "Jianyou").

Similar to the method applied in the chemical pharmaceutical sub-industry, non-target stock companies in the biopharmaceutical sub-industry with significant ATT values that passed the PSM test include Beijing Kawin Technology Share-Holding Co., Ltd (stock code: 688687.sh, hereafter referred to as "Kaiyin"), Sunshine Guojian Pharmaceutical Co., Ltd (stock code: 688336.sh, hereafter referred to as "Sansheng"), Chengdu Olymvax Biopharmaceuticals Inc (stock code: 688319.sh, hereafter referred to as "Oulin"), Sinocelltech Group Limited (stock code: 688520.sh, hereafter referred to as "Shenzhou"), Changchun Bcht Biotechnology Co.(stock code: 688276.sh, hereafter referred to as "Baike"), Liaoning Chengda Biotechnology Co., Ltd (stock code: 688739.sh, hereafter referred to as "Chengda"), Jiangsu GDK Biological Technology Co., Ltd (stock code: 688670.sh, hereafter referred to as "Jindike"), and Bio-Thera Solutions, Ltd (stock code: 688177.sh, hereafter referred to as "Baiaotai"). The stock portfolios are as follows: Junshi-Shenzhou, Junshi-Baike, Junshi-Chengda, Junshi-Jindike; Tiantan-Baiaotai; Jianyou-Kaiyin, Jianyou-Sansheng, Jianyou-Oulin.

The results of the balance hypothesis tests for these eight sets of stocks in the biopharmaceutical sub-industry are summarized in Table S1. As shown in Table S1, the standardized bias (%bias) for all eight sets of stocks is consistently less than 10%. This indicates that the balance effect is quite satisfactory, and there are no significant differences between the matching variables after the matching process. Looking at the intergroup mean T-test, it is evident that there are no significant differences among the stocks after matching, confirming the satisfaction of the balance hypothesis (Table S1).

The visual results of the common support domain test for these eight matched groups are presented in Figure S1, and specific data for the common support domain test for these eight groups are provided in Table S2. The results from Figure S1 and Table S2 demonstrate that the common support hypothesis holds for these eight groups of stocks. The matching process has yielded satisfactory results, meeting the common support assumption.

Table S3 presents the ATT values for these eight groups of stock matching results, along with the corresponding weight values assigned to each successfully matched non-target stock in the prediction model. All of the ATT values for these eight groups of stock matching results surpass 1.96. Remarkably, the ATT value for the Jianyou-Sansheng combination is the highest, reaching 19.71 (Table S3).

The validation results for the IPSO-LSTM model are visually presented in the following figures, comparing stock price prediction outcomes with and without considering

interdependence. Figures S2-1 and S2-2 showcase the stock price prediction results for Junshi. Within Figure S2-1 and S2-2, the orange line represents the true value, the blue line represents predicted results without stock interdependence, and the red and black lines represent predicted results with stock interdependence taken into account. In Figure S2-1, the stock portfolios are Junshi-Shenzhou and Junshi-Baike. In Figure S2-2, the stock portfolios are Junshi-Chengda and Junshi-Jindike.

Additionally, as detailed in Table S4, an evaluation of the performance metrics reveals that, when considering the interdependence effect, Junshi-Shenzhou experiences a decrease of 41.36% in MAPE, 28.30% in RMSE, and 41.28% in MAE, coupled with 2.29% increase in R2. Junshi-Baike sees a decrease of 49.21% in MAPE, 38.95% in RMSE, and 49.00% in MAE, along with 2.95% increase in R2. Junshi-Chengda demonstrates a reduction of 59.16% in MAPE, 46.27% in RMSE, and 59.90% in MAE, with 3.35% increase in R2. Junshi-Jindike exhibits a decrease of 69.63% in MAPE, 62.58% in RMSE, and 70.11% in MAE, coupled with 4.05% increase in R2.

The predictive results for Tiantan and Jianyou are presented in Figures S3, S4-1, and S4-2, along with the relevant evaluation metrics in Table S4. These results provide additional evidence that incorporating matched stock data enhances the accuracy of stock price predictions for the target stocks compared to independent predictions.

Analysis of Stock Price Predictions in the Traditional Chinese Medicine Industry

In the Traditional Chinese Medicine industry, the top five companies based on market capitalization are as follows: Zhangzhou Pientzehuang Pharmaceutical Co.,Ltd (stock code:600436.sh), Beijing Tongrentang Co.,ltd (stock code:600085.sh, hereafter referred to as "Tongrentang"), Guangzhou Baiyunshan Pharmaceutical Holdings Company Limited (stock code:600332.sh), Kangmei Pharmaceutical Co.,Ltd (stock code:600518.sh) and Jumpcan Pharmaceutical Co.,Ltd (stock code:600566.sh, hereafter referred to as "Jichuan").

In the Traditional Chinese Medicine industry, the non-target stock companies that show significant ATT values and pass the PSM test are: Tibet Rhodiola Pharmaceutical Holding Co.,Ltd (stock code:600211.sh, hereafter referred to as "Xizang"), Mayinglong Pharmaceutical Group Co.,Ltd (stock code:600993.sh, hereafter referred to as "Mayinglong"), Tianjin Pharmaceutical Da Ren Tang Group Corporation Limited (stock code:600329.sh, hereafter referred to as "Darentang"). The stock portfolios are as follows: Tongrentang-Xizang; Jichuan-Mayinglong, Jichuan-Darentang.

Table S5 summarizes the results of the balance hypothesis tests for these three groups of matched stocks within the Chinese patent medicine sub-industry. It is evident from Table S5 that the standardized bias (%bias) for all three groups of stocks consistently remains below 10%. This indicates a satisfactory balance effect, implying that there are no significant differences in the matched variables after the matching process. The intergroup mean T-test further validates that these differences among the stocks remain insignificant after matching, affirming the fulfillment of the balance hypothesis (Table S5).

The visual results of the common support domain test for these three matching groups are presented in Figure S5, and you can find specific data related to the common support domain test for these groups in Table S6. The results obtained from Figure S5 and Table S6 affirm the validity of the common support hypothesis for these three stock groups. The matching process has yielded positive results, aligning with the common support assumption.

Table S7 provides information on the ATT values and the weights assigned to each successfully matched non-target stock in the predictive model for these three stock groups. Notably, all three groups exhibit ATT values exceeding 1.96. The highest ATT value is observed in the Jichuan-Mayinglong pair, reaching 9.46.

The validation visualization results for the IPSO-LSTM model are presented in the following figures, comparing the stock price prediction outcomes with and without considering interdependence. Specifically, the stock price prediction results for Tongrentang are depicted in Figure S6. In this figure, the orange line represents the true value, the blue line represents predicted results without stock interdependence, and the black line represents predicted results with stock interdependence taken into account. The stock portfolio is Tongrentang-Xizang. Furthermore, an analysis of the evaluation metrics, as shown in Table S8, indicates that, after considering the interdependence effect, Tongrentang's stock price forecasting experiences a substantial reduction of 12.90% in MAPE, 19.59% in RMSE, and 15.67% in MAE, along with a 0.40% increase in R2.

Moving on to the stock price prediction results for Jichuan, depicted in Figure S7, the orange line represents the true value, the blue line represents predicted results without stock interdependence, and the red and black lines represent predicted results with stock interdependence taken into account. The stock portfolios are Jichuan-Mayinglong and Jichuan-Darentang. As seen in Figure S7, the inclusion of data on these significantly linked stocks significantly improves Jichuan's stock price prediction results. Additionally, an analysis of the evaluation metrics in Table S8 indicates an improvement in the prediction of Jichuan's stock price when considering interdependence effects.
