# Supplementary material for: The application of the propensity score matching method in stock prediction among stocks within the same industry
Source: PeerJ Comput Sci. 2024 Jan 30;10:e1819. doi: 10.7717/peerj-cs.1819 (PMC10909155; doi:10.7717/peerj-cs.1819)
Supplement: Supplemental Information 2 [file peerj-cs-10-1819-s002.pdf]

Appendix 2

Methods

In the initial phase, our primary focus was on adopting Propensity Score Matching (PSM) as the main analytical method. PSM, a technique seeking similarities in observed data, excels in mitigating selection bias in empirical studies. However, to ensure research robustness and comprehensive result validation, this paper introduces another analytical approach—ridge regression analysis—for comparison. The choice of ridge regression is motivated by multicollinearity issues in the stock data, involving multiple stocks within the same industry. Ridge regression, addressing multicollinearity problems through regularization terms, enhances model stability and interpretability, making it a suitable choice for accurately revealing interdependence features among industry stocks.

Considering the unique nature of the IPSO-LSTM model—a fusion of the chaos particle swarm optimization algorithm and Long Short-Term Memory (LSTM) network—optimizing LSTM parameters enables more effective capture of non-linear relationships in stock data. We employ IPSO-LSTM as a validation method to minimize model-induced errors, thereby maximizing the representation of errors arising from the propensity score matching method in studying interdependence. This aims to provide a more accurate reflection of stock interdependence within the same industry. However, it is essential to acknowledge that optimizing this model may influence our judgment and the chosen validation method.

To comprehensively evaluate the comparison between propensity score matching and ridge regression analysis, this study introduces the baseline LSTM model, with parameters unoptimized through IPSO. The baseline LSTM model allows for a direct comparison of the effectiveness of propensity score matching and ridge regression analysis in studying interdependence without additional optimization. This ensures a more comprehensive and objective assessment of the two methods.

Results

In pharmaceutical chemistry, Hengrui uses the propensity score matching method to identify Huana as a notably significant stock for the Average Treatment Effect on the Treated (ATT). Employing ridge regression analysis, the independent variables include the closing prices of all stocks in the chemical pharmaceutical sector, excluding Hengrui's stock. The dependent variable is the closing price of Hengrui's stock. For comparative validation, the stock with the highest correlation coefficient, Zhangjiang, is chosen. The correlation coefficient for Zhangjiang is 0.36, as shown in Table S9. Subsequently, an experimental validation of stock combinations,

specifically Hengrui-Huana and Hengrui-Zhangjiang, is conducted concerning Hengrui's target stocks. The correlation coefficient, acting as the weight of the selected stocks in the predictive model, is integral to this analysis.

The impact of these methods on predicting target stock prices was verified using IPSO-LSTM and LSTM models. Under the IPSO-LSTM model, results for Hengrui-Huana and Hengrui-Zhangjiang are depicted in Figure S8-1. In Figure S8-1, "true" represents the actual stock prices in the Hengrui test set, while "Zhangjiang" signifies the predictive outcomes combining Hengrui with Zhangjiang stock data. Zhangjiang stock data demonstrates the highest correlation coefficient in the ridge regression analysis. "Huana" indicates the predictive outcomes when combining Hengrui with Huana data. Examining Figure S8-1 reveals that incorporating Huana into the predictive results yields superior performance compared to the inclusion of Zhangjiang. Assessment metrics in Table S10 under the IPSO-LSTM model show that, with the inclusion of Huana stock, Hengrui stock price predictions exhibit reductions of 22.73%, 18.85%, and 23.80% in MAPE, RMSE, and MAE, respectively, compared to the Hengrui-Zhangjiang predictions. Additionally, R2 shows an increase of 0.76%.

Results under the baseline LSTM model for Hengrui-Huana and Hengrui-Zhangjiang are presented in Figure S8-2. Similar observations are made, where the inclusion of Huana in the prediction produces superior results compared to Zhangjiang. Evaluation metrics in Table S10 further support this, with decreased MAPE, RMSE, and MAE, and increased R^2 after considering Huana 's stock.

Similarly, by observing the evaluation metrics data in Table S10, it is intuitively clear that the prediction results of IPSO-LSTM are superior to those of the baseline LSTM for the same stock combinations.

In the application of propensity score matching analysis by Fuxing, two stocks with notably significant Average Treatment Effects on the Treated (ATT) were identified—namely, Huana and Hengrui—where Huana demonstrated the maximum ATT. For comparative validation, the two stocks with the highest correlation coefficients obtained from ridge regression analysis were selected: Borui and Yaoming. Borui exhibited a correlation coefficient of 0.33, while Yaoming showed a correlation coefficient of 0.30. Therefore, for the target stocks of Fuxing, an experimental validation of stock combinations—specifically Fuxing-Borui and Fuxing-Huana, and Fuxing- Yaoming and Fuxing -Hengrui—was conducted. The correlation coefficient outcomes are detailed in Table S9. Under the IPSO-LSTM model, the results for Fuxing-Borui and Fuxing-Huana are depicted in Figure S9-1, while the outcomes under the benchmark LSTM model are illustrated in Figure S9-2. Specific model evaluation indicator data are shown in Table

S11. In the IPSO-LSTM model, compared to ridge regression analysis, the MAPE, RMSE, and MAE for Fuxing-Huana decreased by 46.67%, 35.08%, and 45.18%, respectively, with a concurrent increase of 1.56% in R2. In the LSTM model, in contrast to Fuxing -Borui, Fuxing-Huana exhibited reductions of 3.43%, 8.63%, and 4.15% in MAPE, RMSE, and MAE, respectively, along with an increase of 3.74% in R2. Similarly, Results under the IPSO-LSTM model for Fuxing- Yaoming and Fuxing -Hengrui are shown in Figure S9-3, and under the baseline LSTM model in Figure S9-4. Evidently, the propensity score matching method's stock pairs yield superior predictive results compared to ridge regression analysis.

Renfu, utilizing the propensity score matching method, identified Chengdu with a significant ATT. In ridge regression analysis, the stock with the highest correlation coefficient was Hengrui, with a coefficient of 0.47. Experimental verification for Renfu's target stocks compared stock combinations Renfu-Chengdu and Renfu-Hengrui. Results under the IPSO-LSTM model for Renfu-Chengdu and Renfu-Hengrui are shown in Figure S10-1, and under the baseline LSTM model in Figure S10-2. Specific model evaluation indicator data are shown in Table S12. Consistent with previous observations, the figures highlight the propensity score matching method's stock pairs exhibiting superior predictive results compared to ridge regression analysis.

Huahai used the propensity score matching method to select Renfu with a significant ATT. Ridge regression analysis pointed to Haizheng, with a correlation coefficient of 0.54. Experimental verification for Huahai's target stocks compared stock combinations Huahai-Renfu and Huahai-Haizheng. Results under the IPSO-LSTM model for Huahai-Renfu and Huahai-Haizheng are shown in Figure S11-1, and under the baseline LSTM model in Figure S11-2. Specific model evaluation indicator data are shown in Table S13. As observed in previous cases, the figures illustrate the propensity score matching method's stock pairs outperforming ridge regression analysis in predicting stock prices.

Conclusion

This appendix primarily showcases results in the chemical pharmaceutical industry, and similar validation methods in other industries have yielded consistent conclusions. These findings underscore the advantages of propensity score matching in verifying stock interdependence. Simultaneously, they confirm that IPSO-LSTM excels in stock price prediction compared to the baseline LSTM.
