# Supplementary figures and images for: The application of the propensity score matching method in stock prediction among stocks within the same industry
Source: PeerJ Comput Sci. 2024 Jan 30;10:e1819. doi: 10.7717/peerj-cs.1819 (PMC10909155; doi:10.7717/peerj-cs.1819)

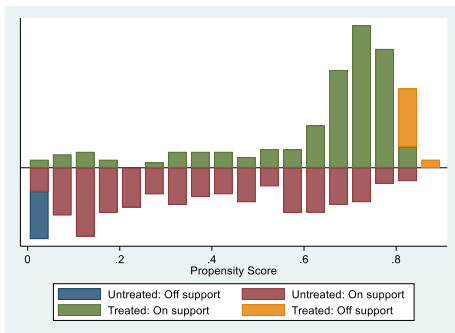

(a)

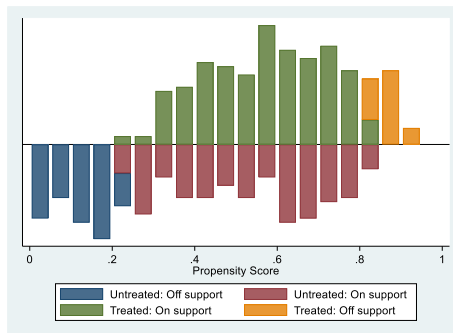

(b)

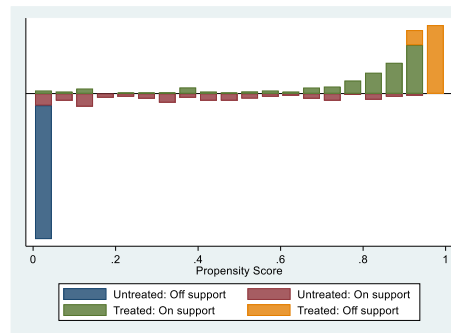

(c)

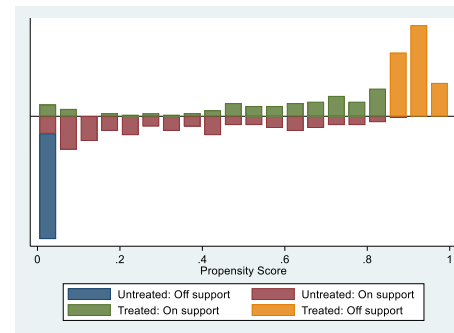

(d)

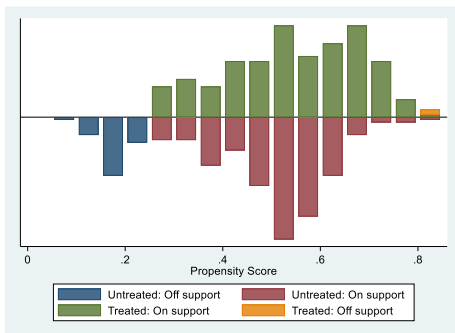

(e)

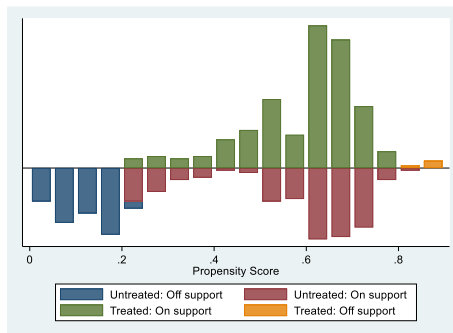

(f)

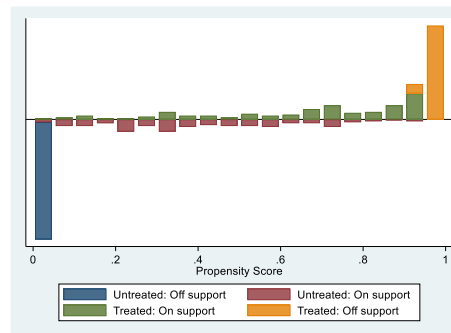

(g)

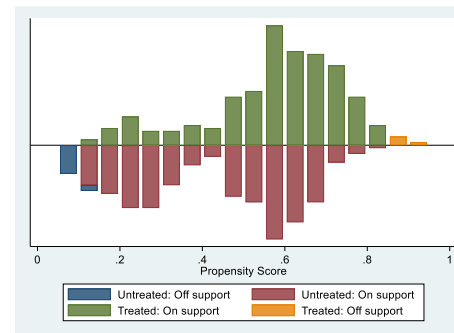

(h)

Supplement: Supplemental Information 3 — (a)Junshi-Shenzhou, (b)Junshi-Baike, (c)Junshi-chengda, (d)Junshi-Jindike, (e)Tiantan-Baiaotai, (f)Jianyou-Kaiyin, (g)Jianyou-Sansheng, (h)Jianyou-Oulin. [file peerj-cs-10-1819-s003.pdf]

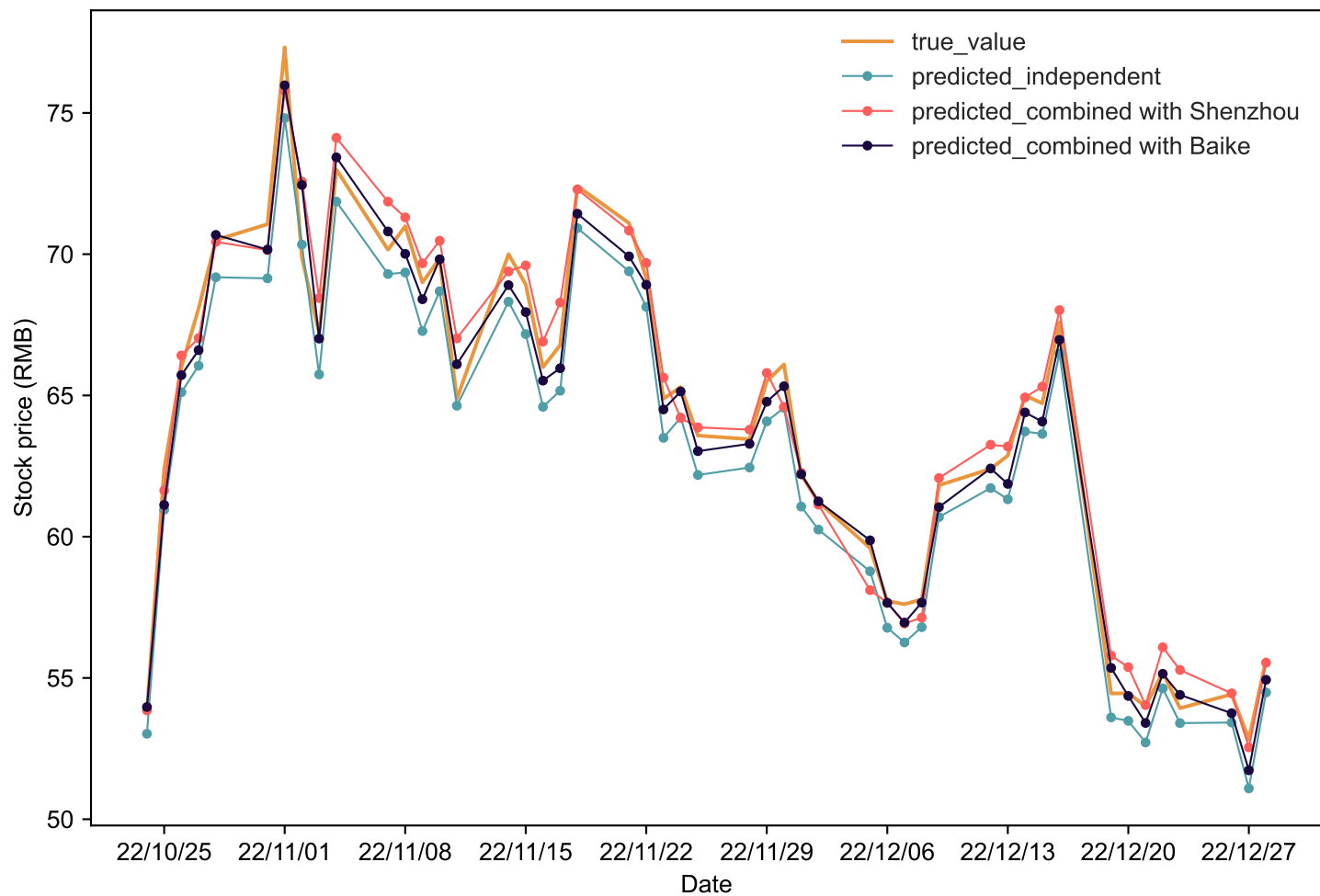

Supplement: Supplemental Information 4 — Note: True value is depicted by the orange line, predicted results without stock interdependence are represented by the blue line, and predicted results with stock interdependence taken into account are illustrated by the black line and the red line. The stock portfolios are Junshi-Shenzhou and Junshi-Baike. [file peerj-cs-10-1819-s004.pdf]

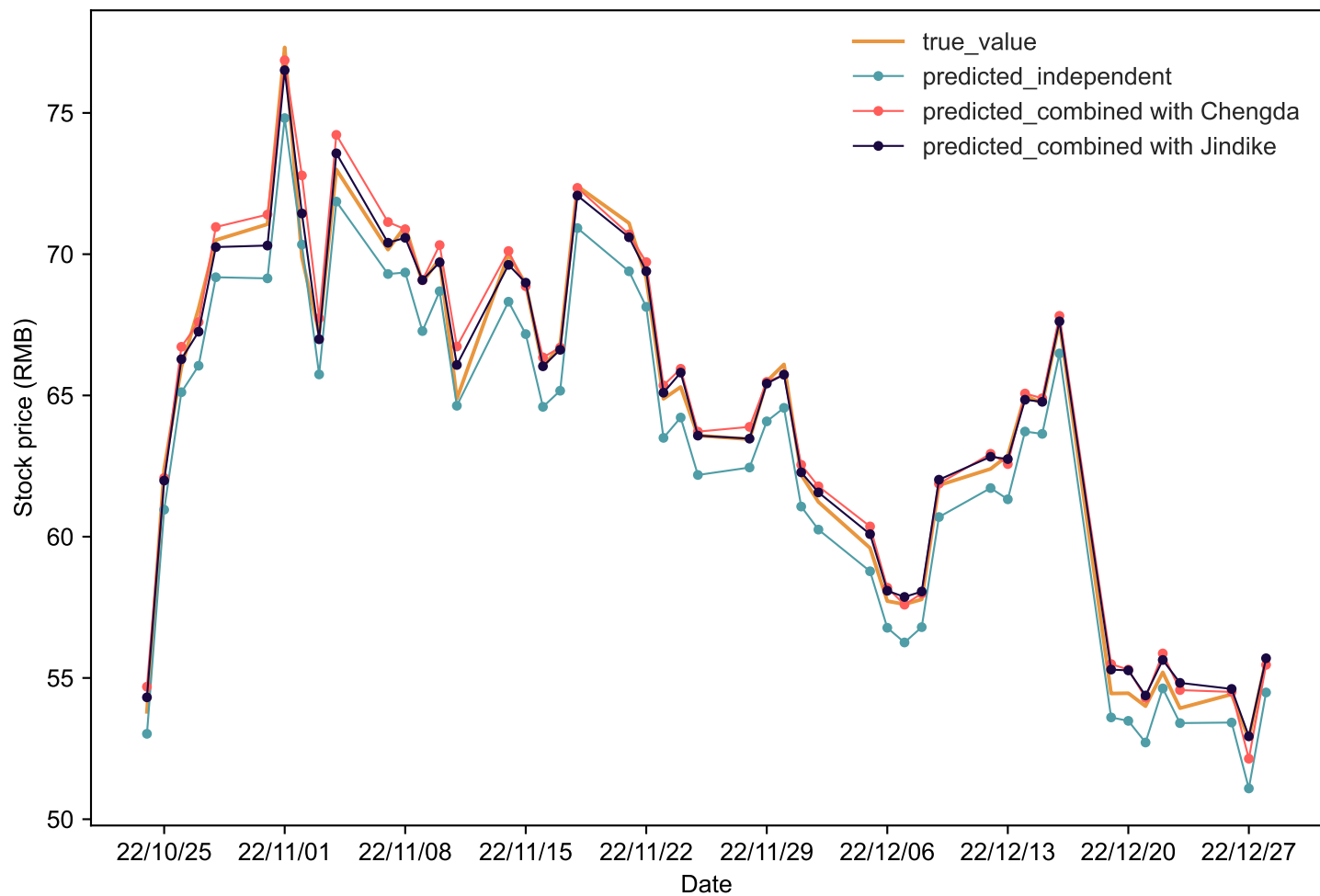

Supplement: Supplemental Information 5 — Note: True value is depicted by the orange line, predicted results without stock interdependence are represented by the blue line, and predicted results with stock interdependence taken into account are illustrated by the black line and the red line. The stock portfolios are Junshi-Chengda and Junshi-Jindike. [file peerj-cs-10-1819-s005.pdf]

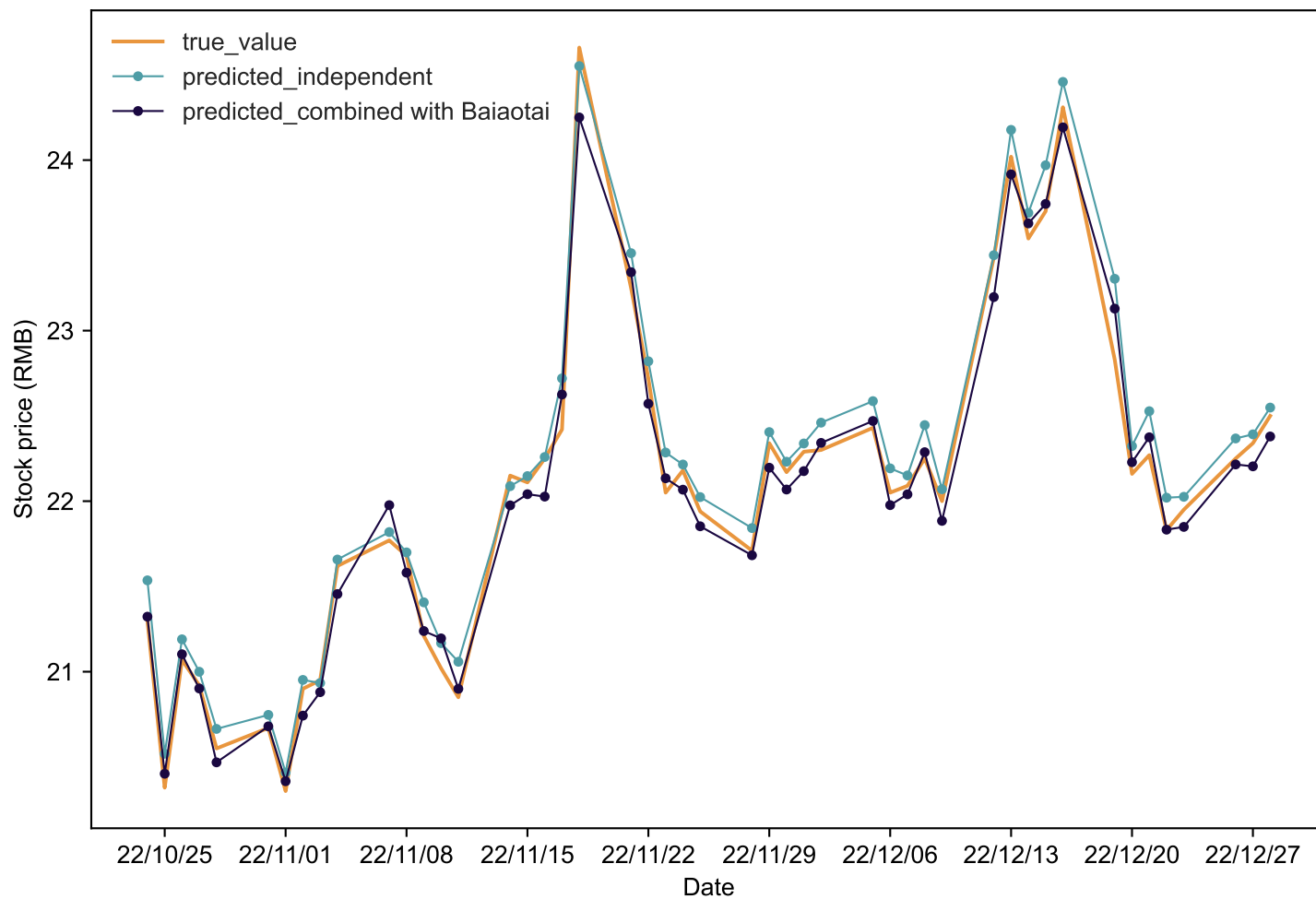

Supplement: Supplemental Information 6 — Note: True value is depicted by the orange line, predicted results without stock interdependence are represented by the blue line, and predicted results with stock interdependence taken into account are illustrated by the black line. [file peerj-cs-10-1819-s006.pdf]

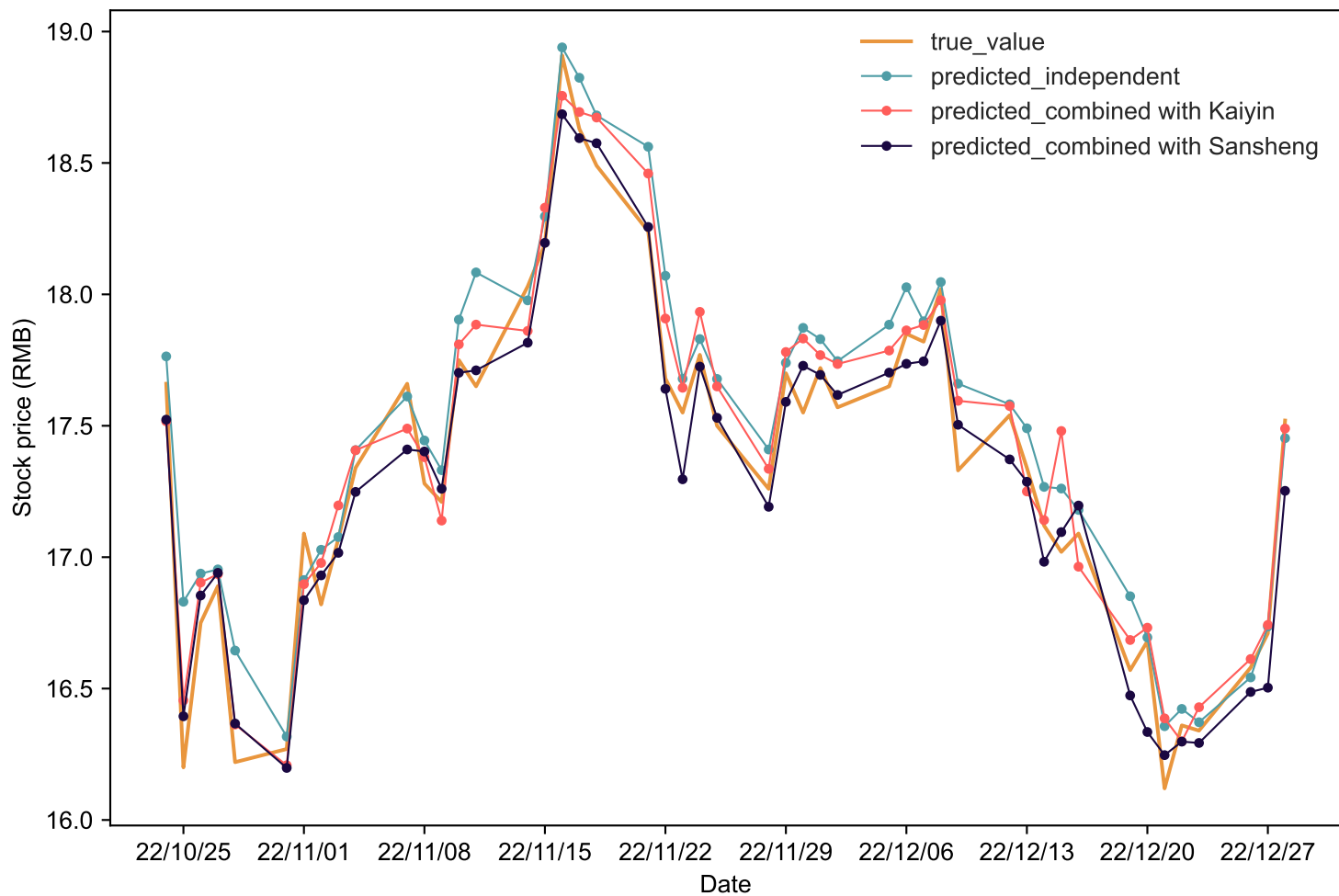

Supplement: Supplemental Information 7 — Note: True value is depicted by the orange line, predicted results without stock interdependence are represented by the blue line, and predicted results with stock interdependence taken into account are illustrated by the black line and the red line. The stock portfolios are Jianyou-Kaiyin and Jianyou-Sansheng. [file peerj-cs-10-1819-s007.pdf]

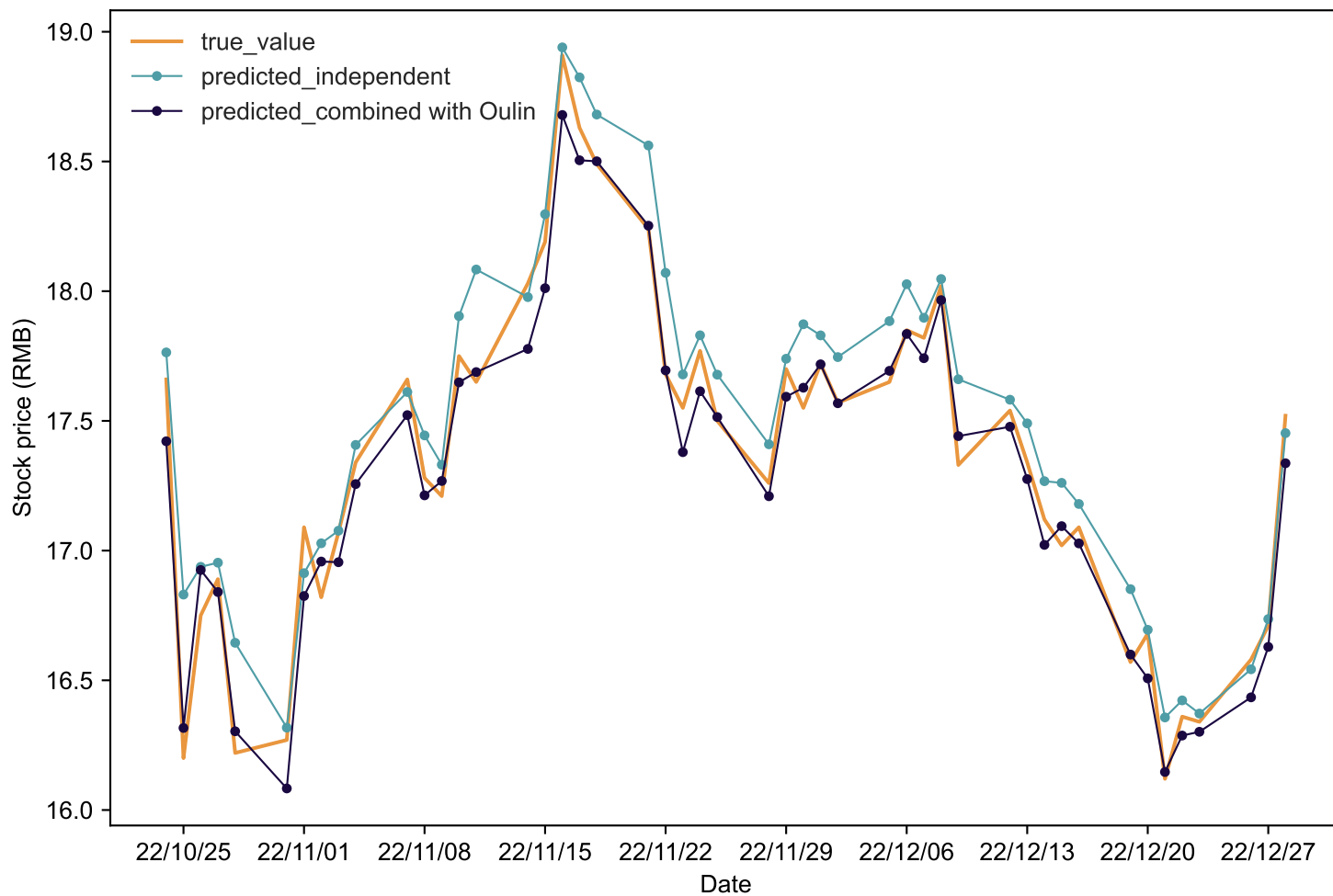

Supplement: Supplemental Information 8 — Note: True value is depicted by the orange line, predicted results without stock interdependence are represented by the blue line, and predicted results with stock interdependence taken into account are illustrated by the black line. The stock portfolios is Jianyou-Oulin. [file peerj-cs-10-1819-s008.pdf]

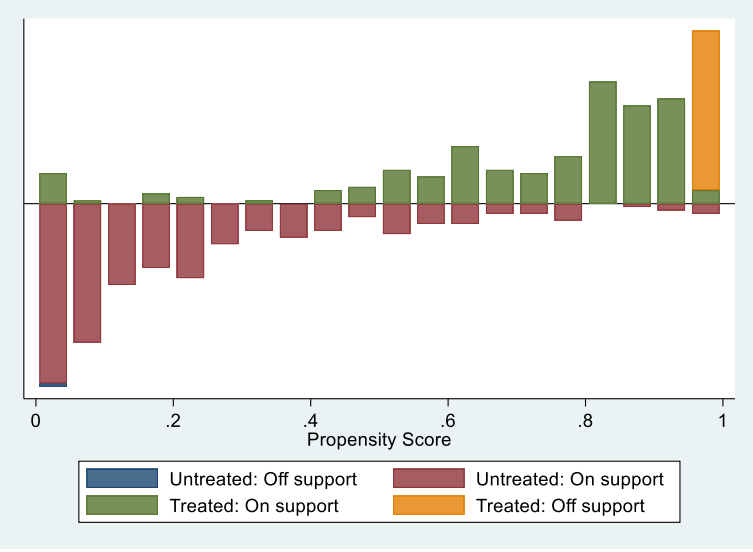

(a)

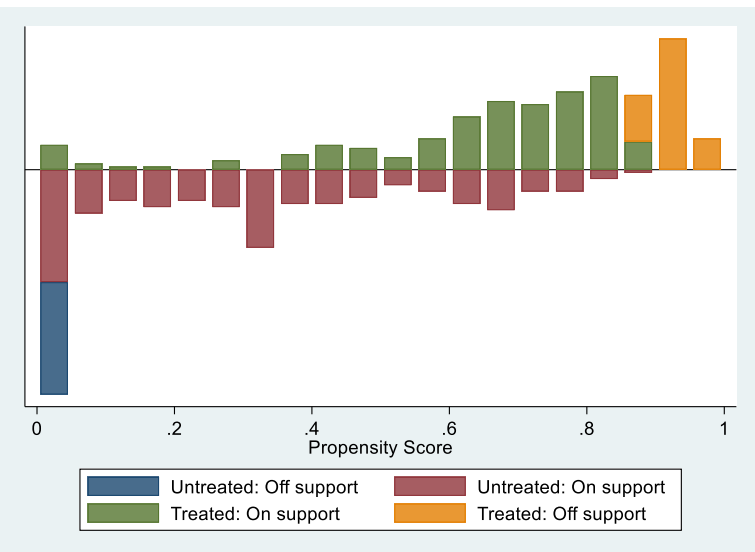

(b)

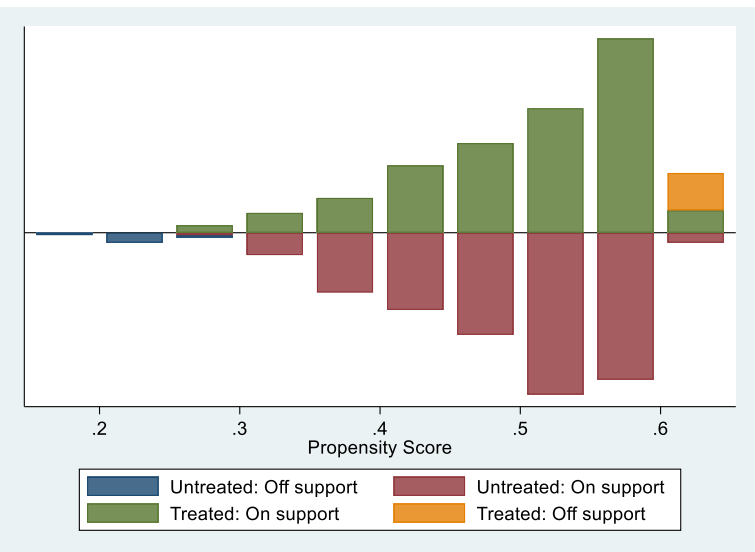

(c)

Supplement: Supplemental Information 9 — (a) Tongrentang-Xizang, (b) Jichuan-Mayinglong, (c) Jichuan-Darentang [file peerj-cs-10-1819-s009.pdf]

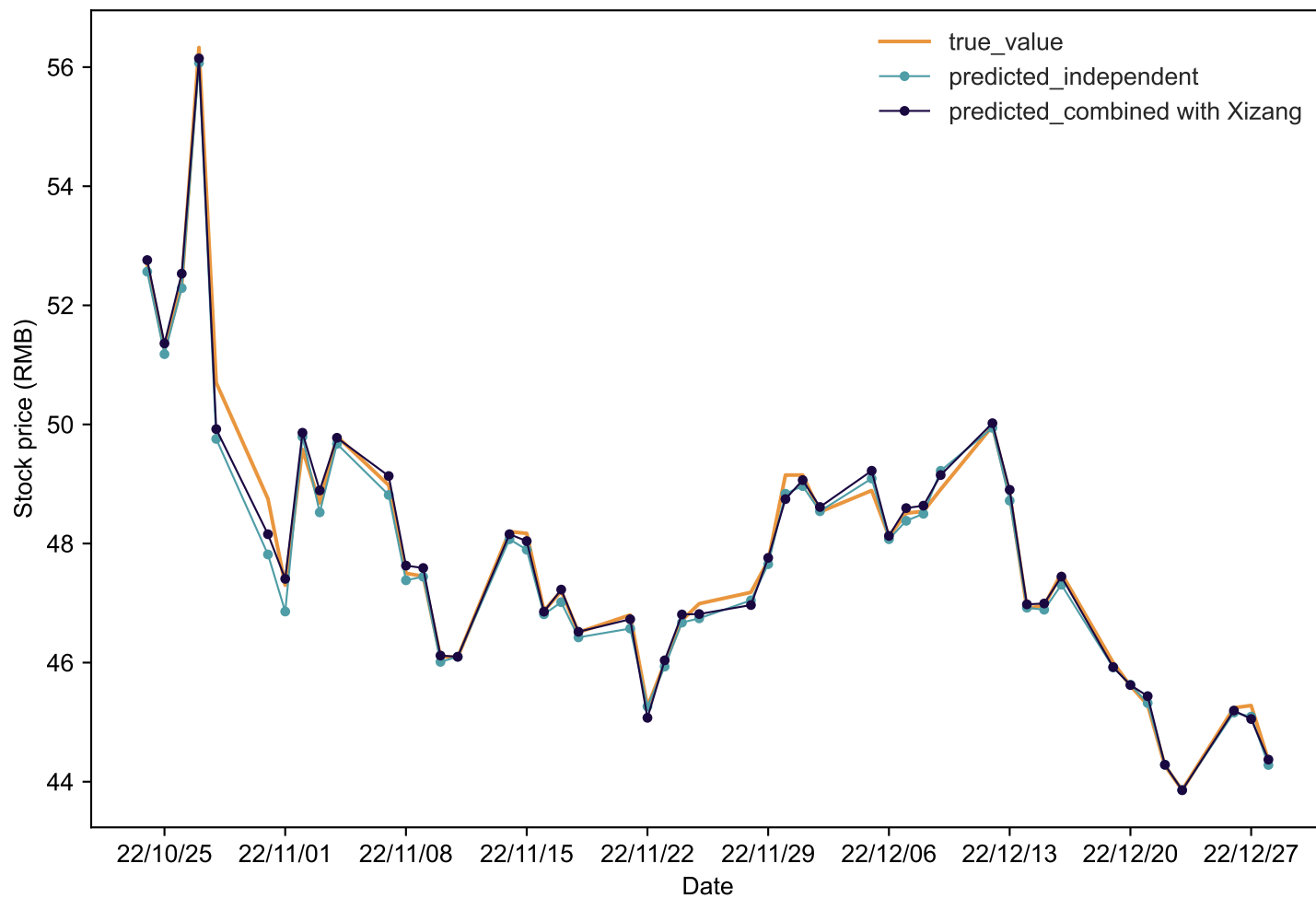

Supplement: Supplemental Information 10 — Note: True value is depicted by the orange line, predicted results without stock interdependence are represented by the blue line, and predicted results with stock interdependence taken into account are illustrated by the black line. [file peerj-cs-10-1819-s010.pdf]

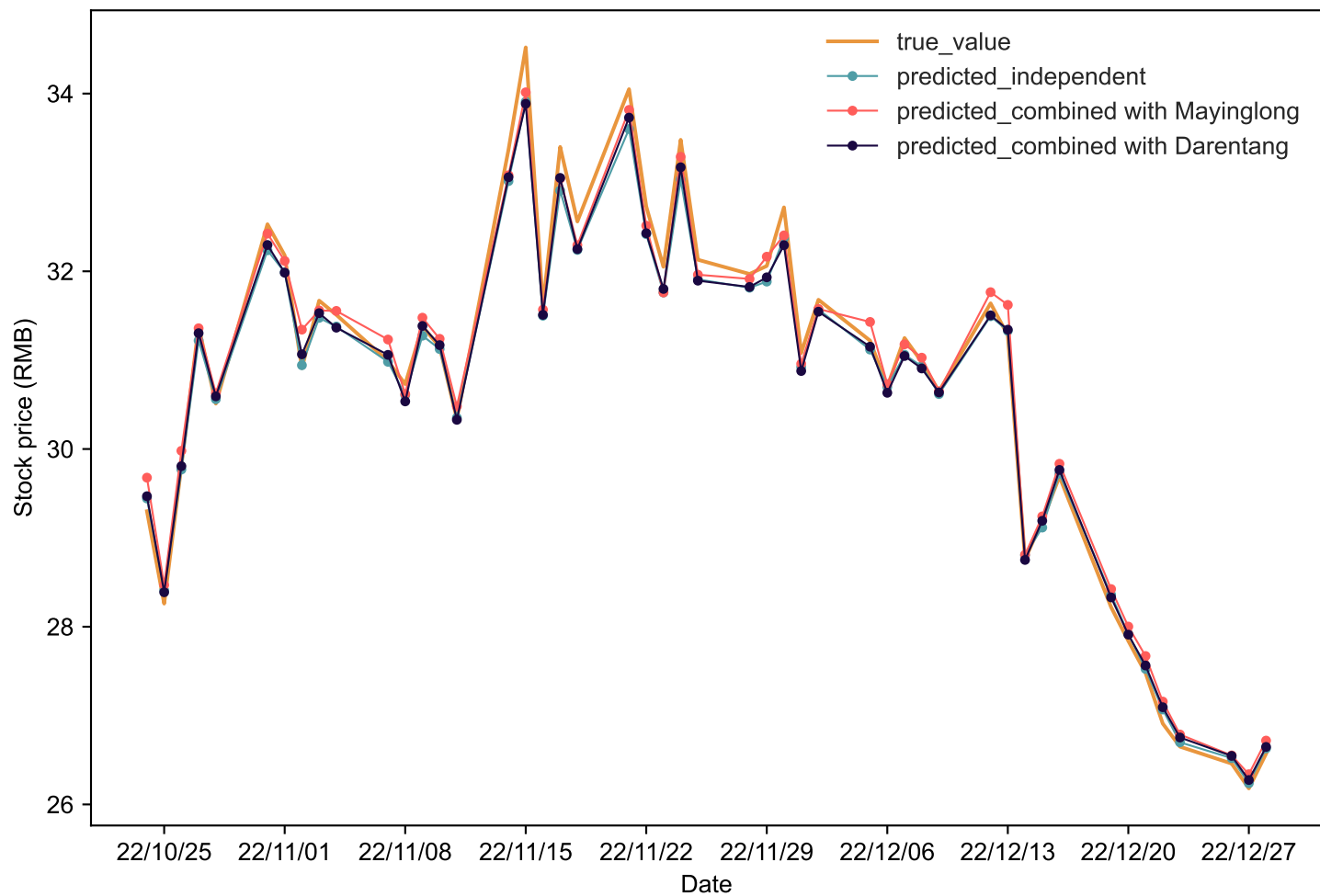

Supplement: Supplemental Information 11 — Note: True value is depicted by the orange line, predicted results without stock interdependence are represented by the blue line, and predicted results with stock interdependence taken into account are illustrated by the black line and the red line. [file peerj-cs-10-1819-s011.pdf]

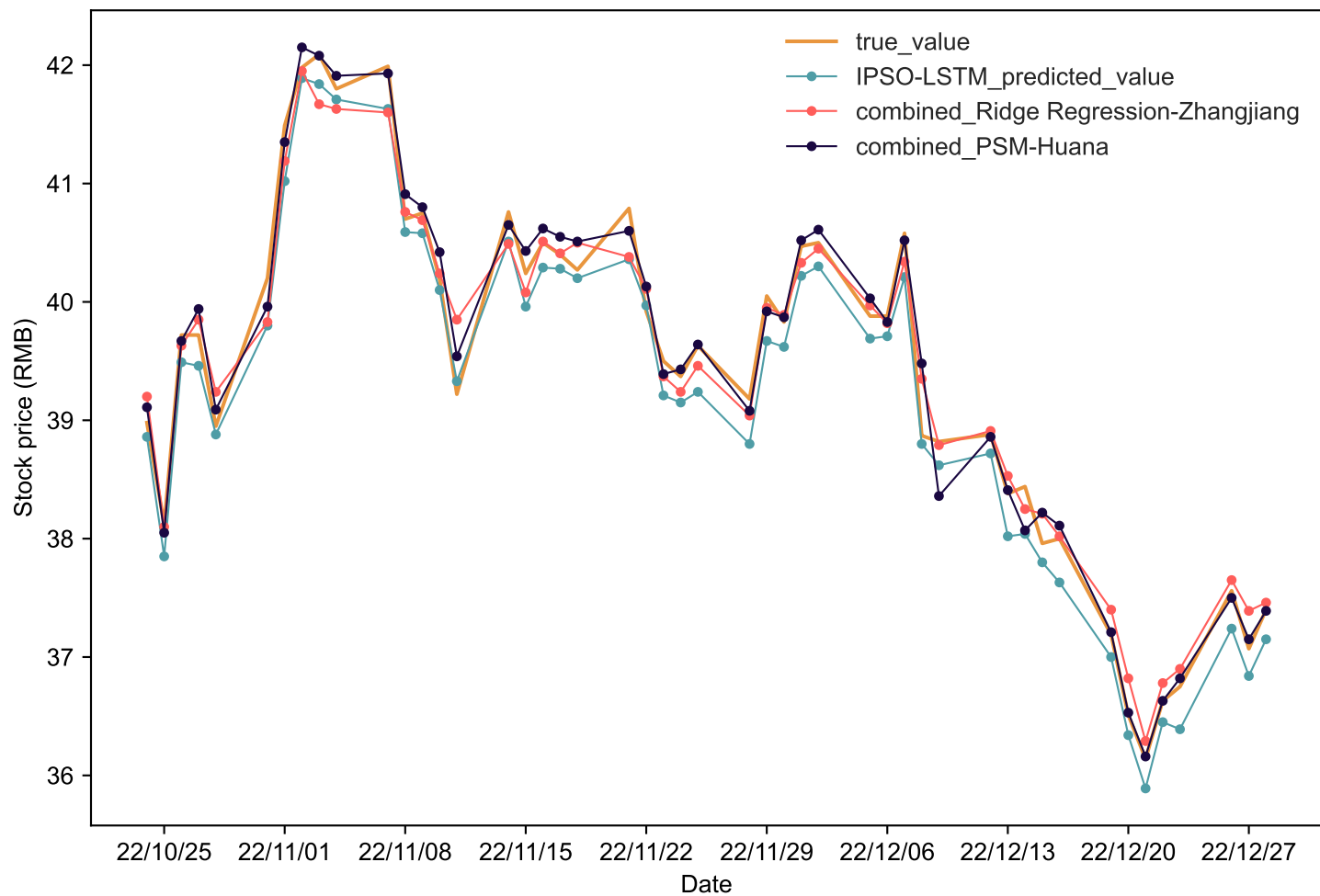

Supplement: Supplemental Information 12 — Note: True value is depicted by the orange line, the IPSO-LSTM model predictions without stock interdependencice are represented by the blue line, the red line indicates the IPSO-LSTM predictions considering ridge regression, and the IPSO-LSTM predictions considering PSM are illustrated by the black line. [file peerj-cs-10-1819-s012.pdf]

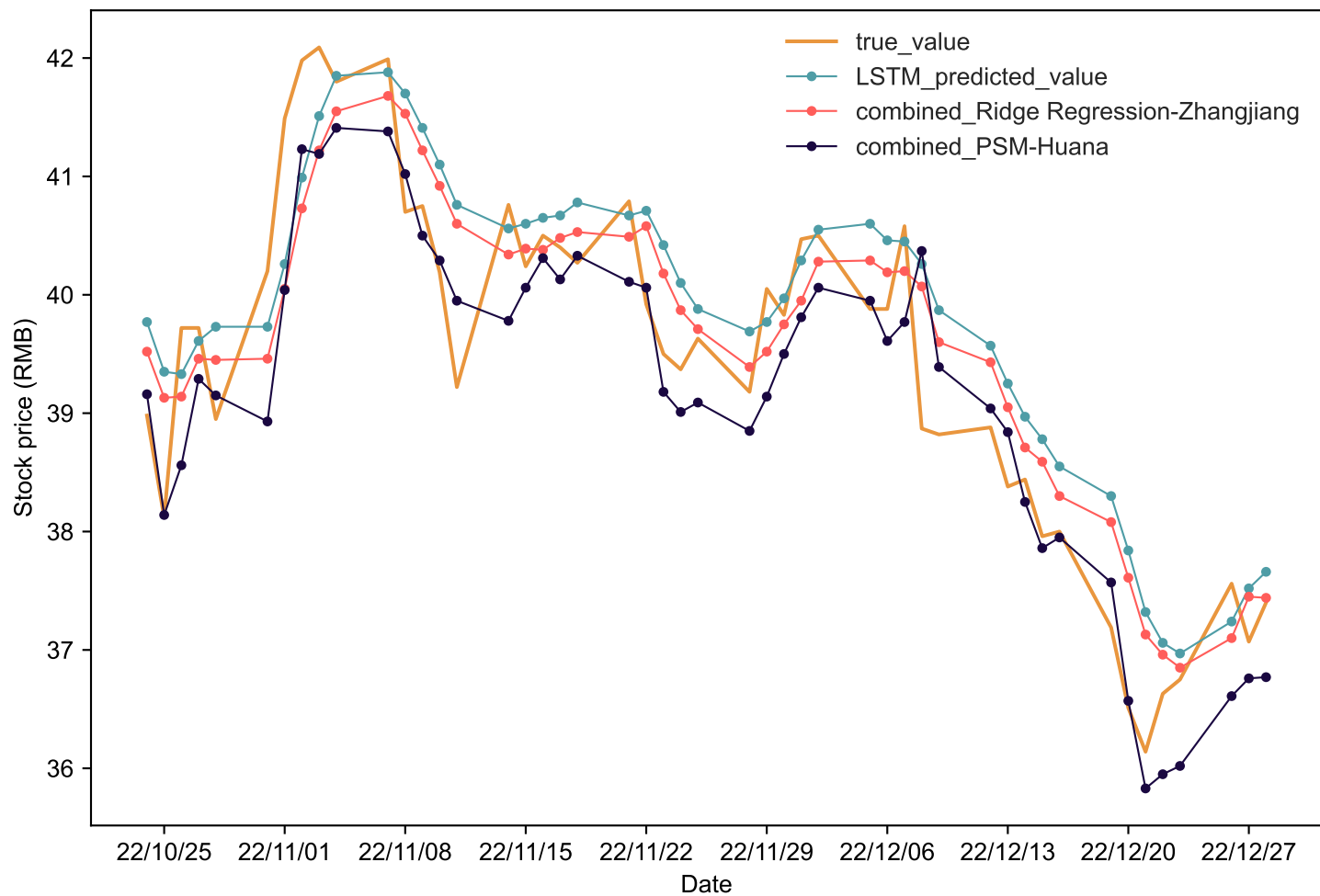

Supplement: Supplemental Information 13 — Note: True value is depicted by the orange line, the LSTM model predictions without stock interdependencice are represented by the blue line, the red line indicates the LSTM predictions considering ridge regression, and the LSTM predictions considering PSM are illustrated by the black line. [file peerj-cs-10-1819-s013.pdf]

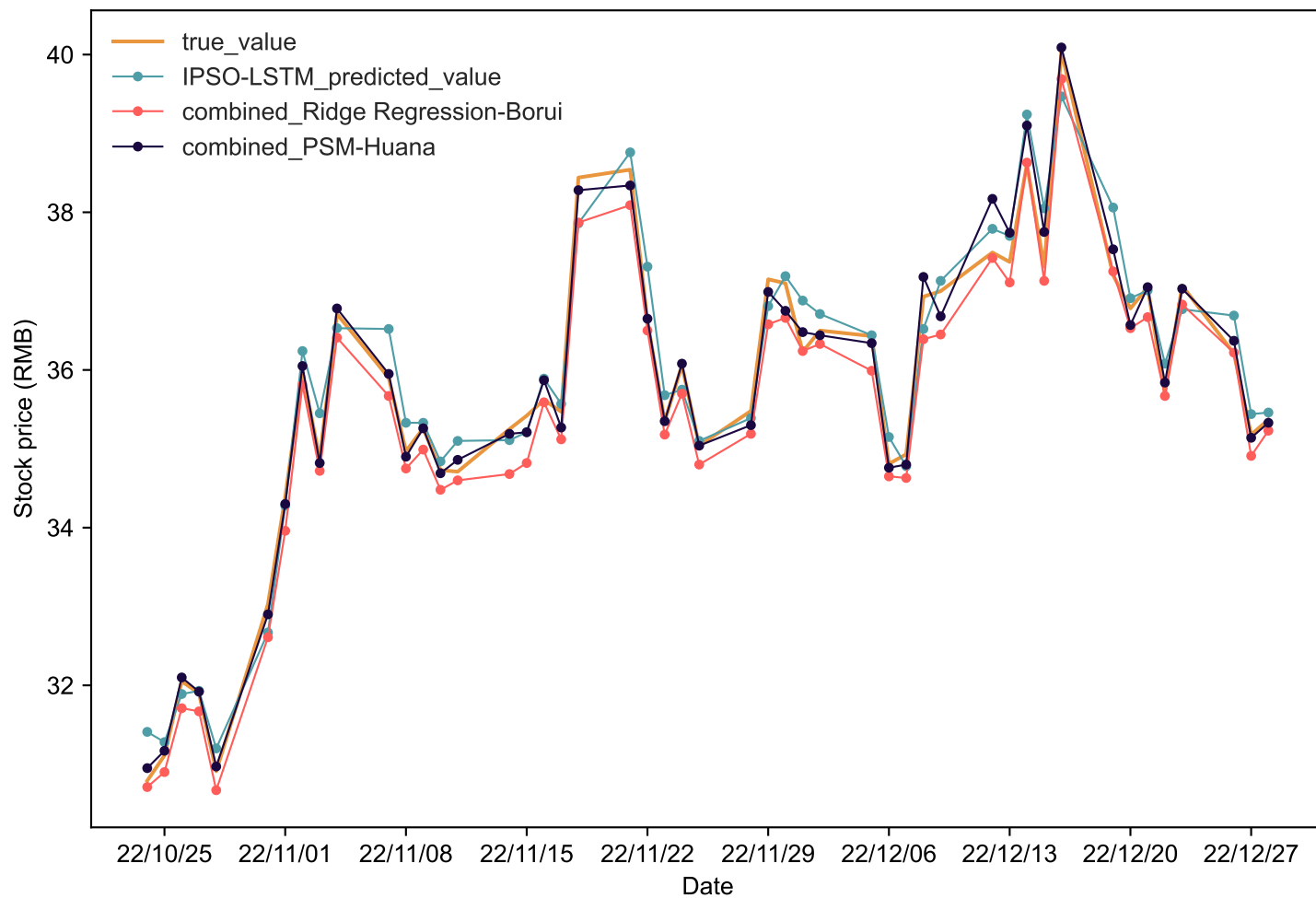

Supplement: Supplemental Information 14 — Note: True value is depicted by the orange line, the IPSO-LSTM model predictions without stock interdependencice are represented by the blue line, the red line indicates the IPSO-LSTM predictions considering ridge regression, and the IPSO-LSTM predictions considering PSM are illustrated by the black line. The stock combinations are Fuxing-Borui and Fuxing-Huana. [file peerj-cs-10-1819-s014.pdf]

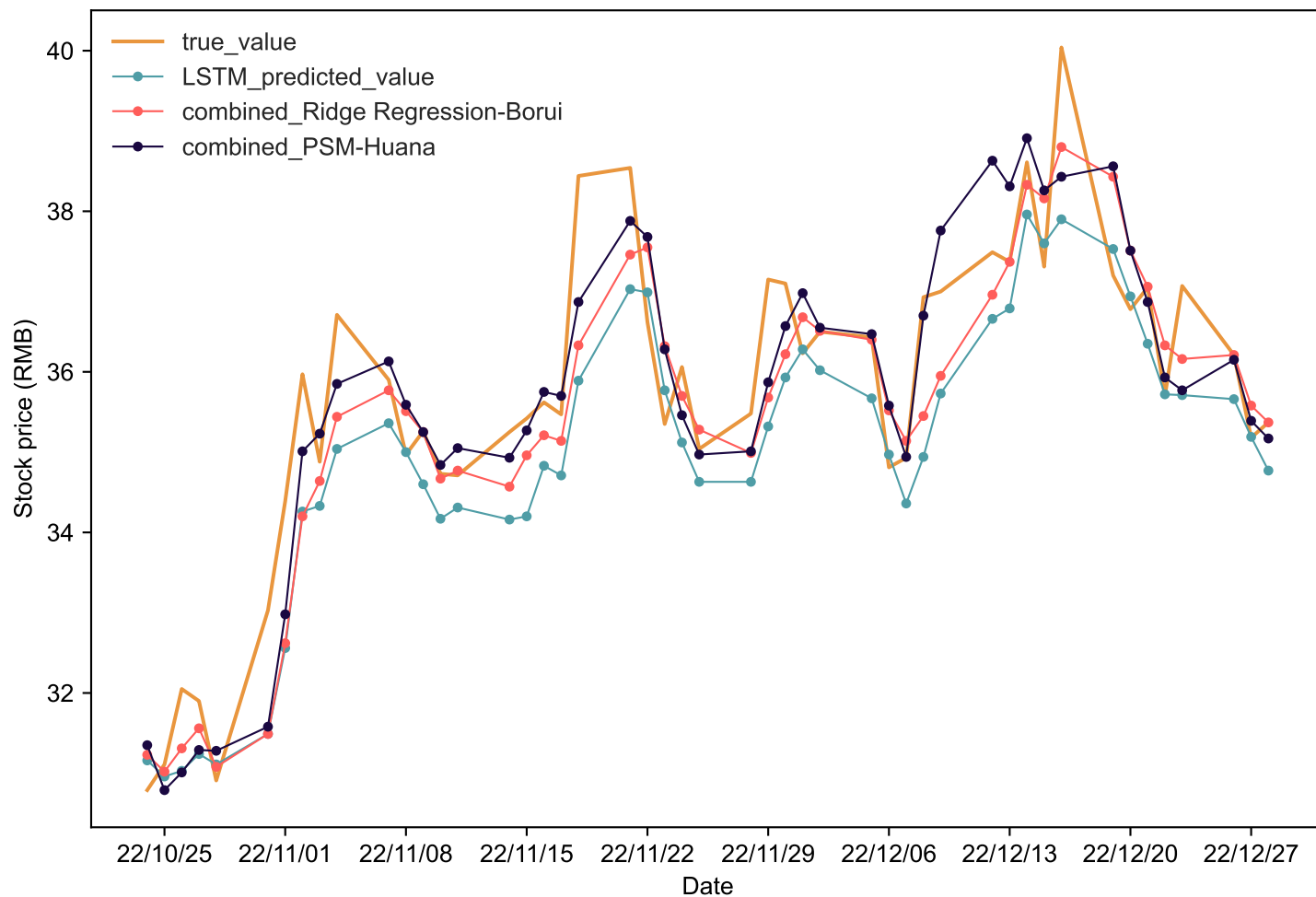

Supplement: Supplemental Information 15 — Note: True value is depicted by the orange line, the LSTM model predictions without stock interdependencice are represented by the blue line, the red line indicates the LSTM predictions considering ridge regression, and the LSTM predictions considering PSM are illustrated by the black line. The stock combinations are Fuxing-Borui and Fuxing-Huana. [file peerj-cs-10-1819-s015.pdf]

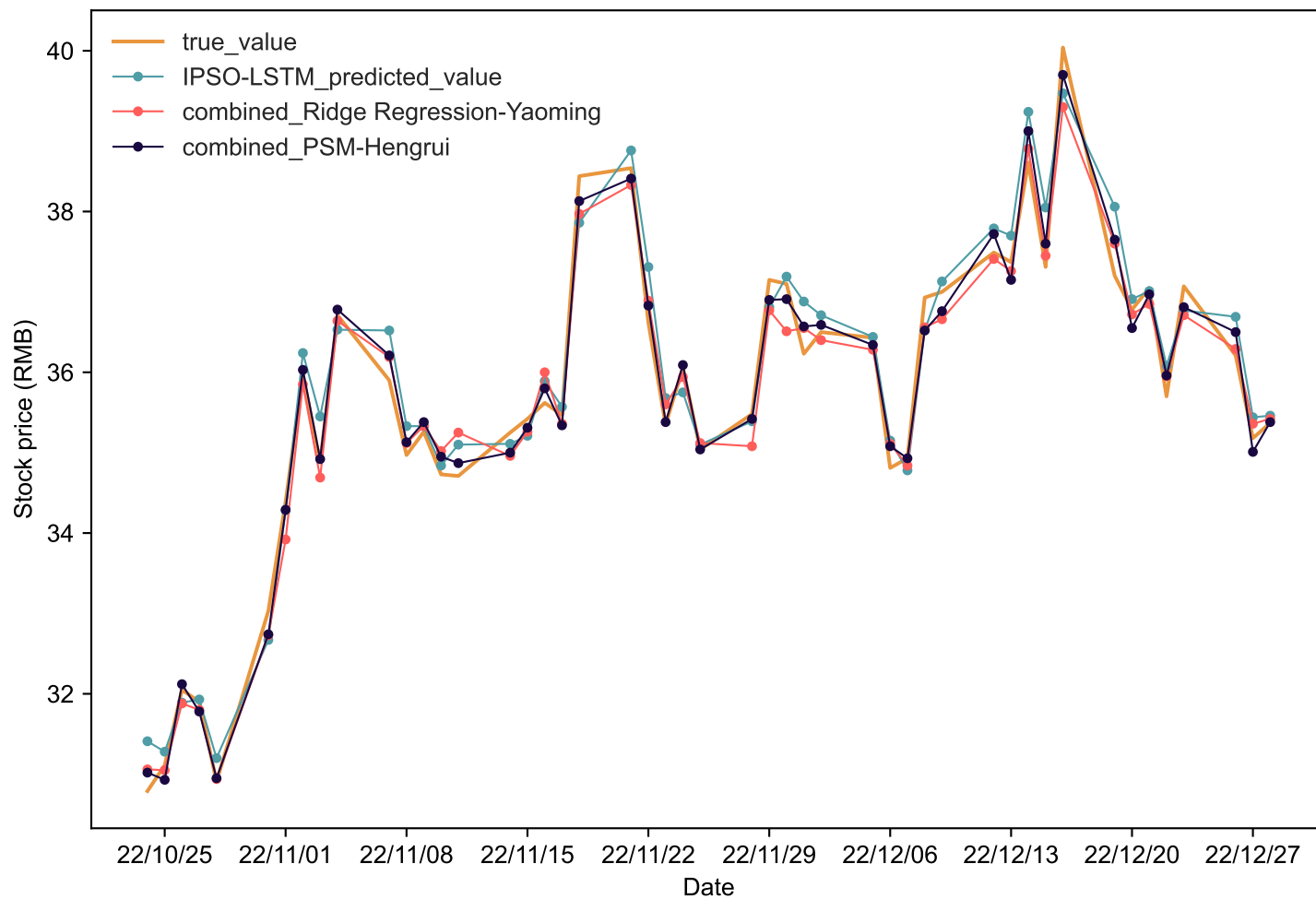

Supplement: Supplemental Information 16 — Note: True value is depicted by the orange line, the IPSO-LSTM model predictions without stock interdependencice are represented by the blue line, the red line indicates the IPSO-LSTM predictions considering ridge regression, and the IPSO-LSTM predictions considering PSM are illustrated by the black line.The stock combinations are Fuxing-Yaoming and Fuxing-Hengrui. [file peerj-cs-10-1819-s016.pdf]

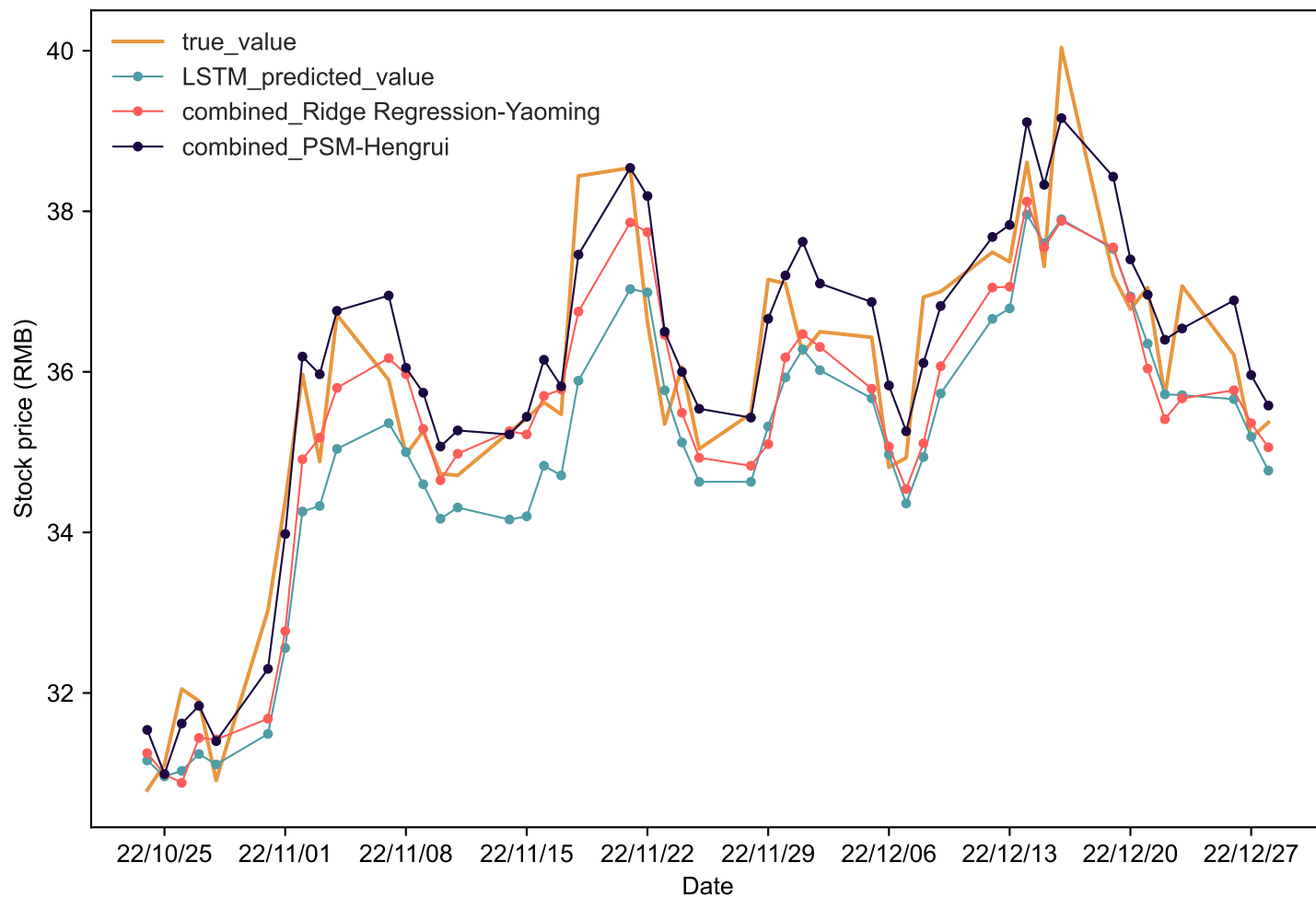

Supplement: Supplemental Information 17 — Note: True value is depicted by the orange line, the LSTM model predictions without stock interdependencice are represented by the blue line, the red line indicates the LSTM predictions considering ridge regression, and the LSTM predictions considering PSM are illustrated by the black line.The stock combinations are Fuxing-Yaoming and Fuxing-Hengrui. [file peerj-cs-10-1819-s017.pdf]

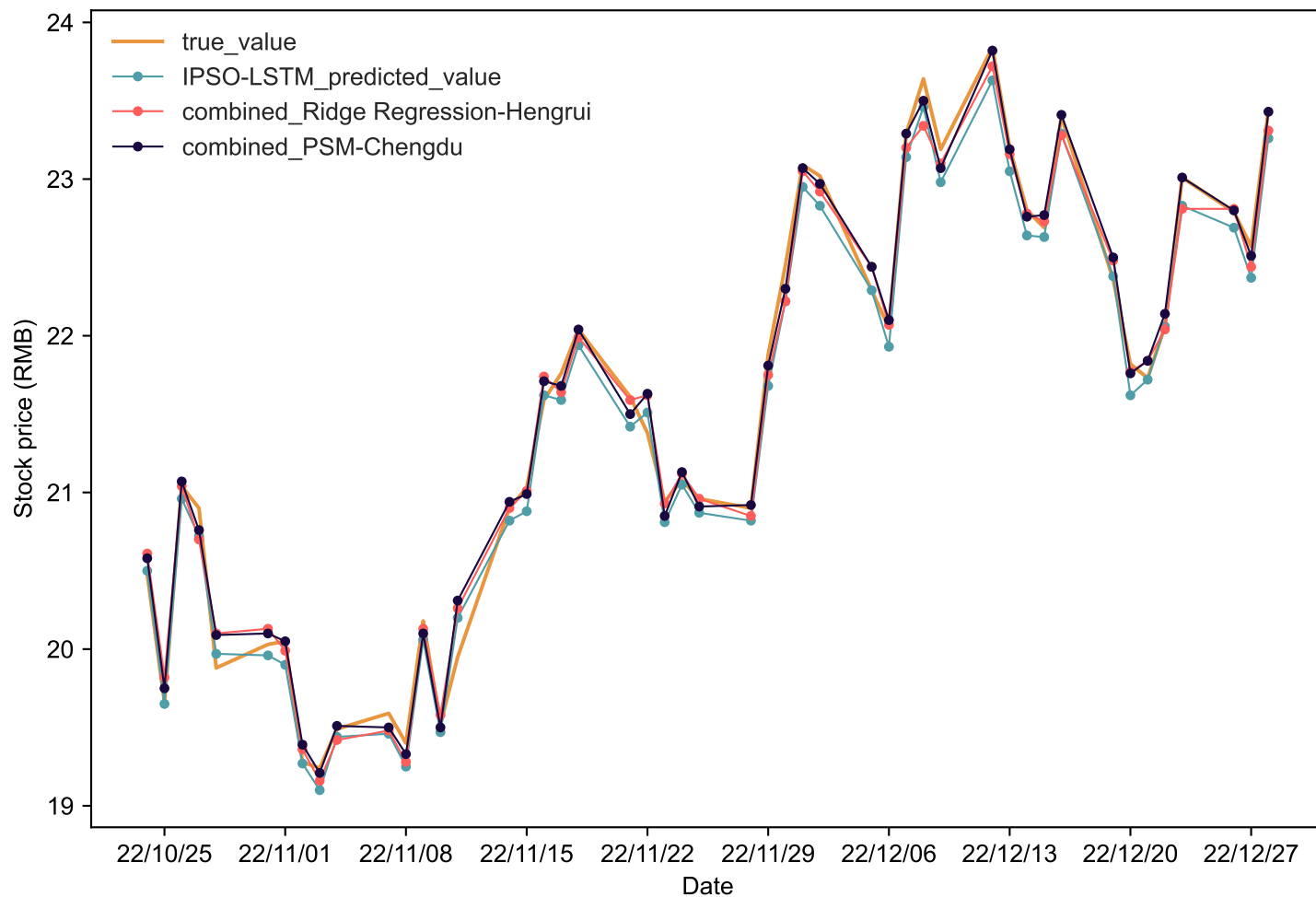

Supplement: Supplemental Information 18 — Note: True value is depicted by the orange line, the IPSO-LSTM model predictions without stock interdependencice are represented by the blue line, the red line indicates the IPSO-LSTM predictions considering ridge regression, and the IPSO-LSTM predictions considering PSM are illustrated by the black line. [file peerj-cs-10-1819-s018.pdf]

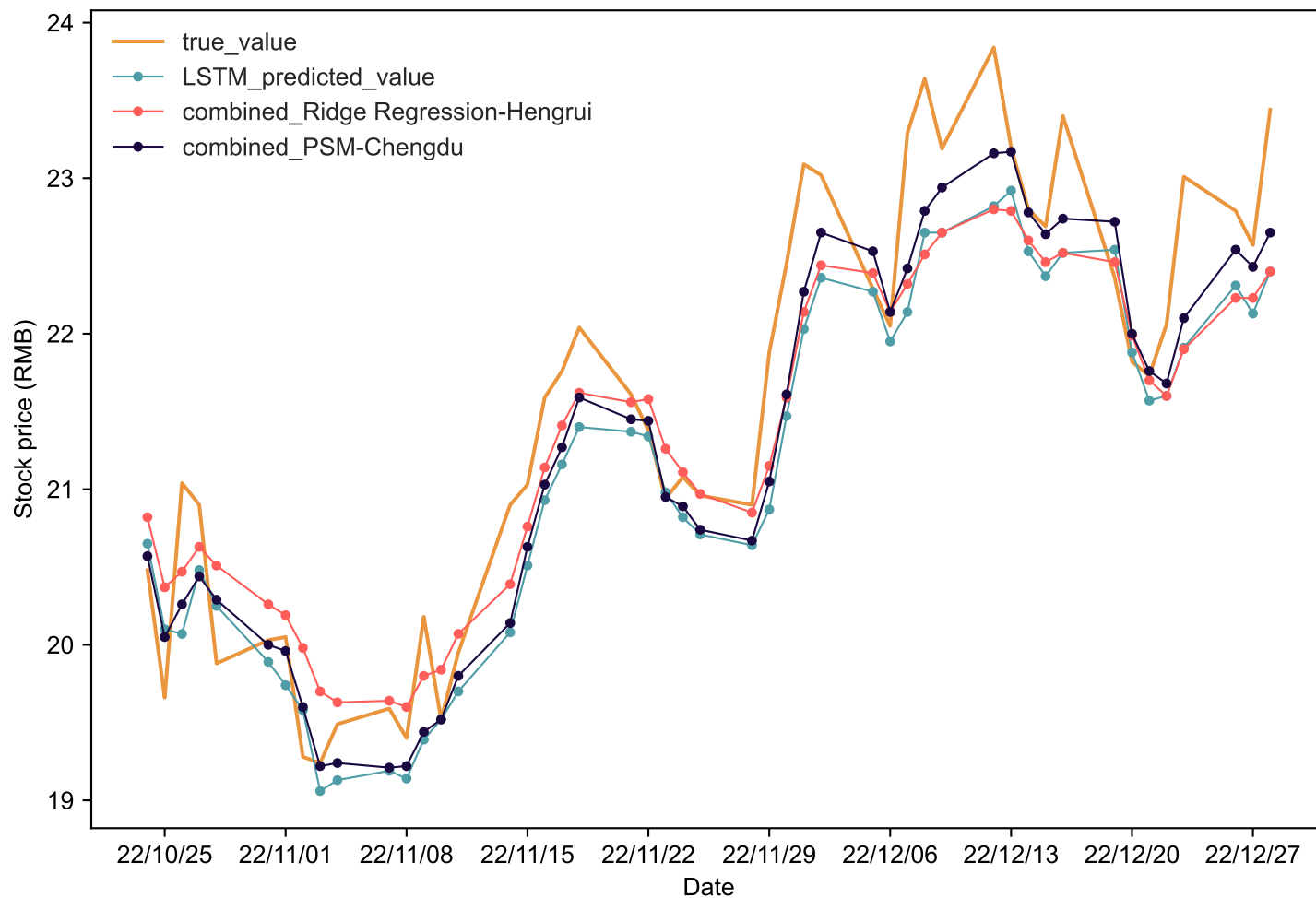

Supplement: Supplemental Information 19 — Note: True value is depicted by the orange line, the LSTM model predictions without stock interdependencice are represented by the blue line, the red line indicates the LSTM predictions considering ridge regression, and the LSTM predictions considering PSM are illustrated by the black line. [file peerj-cs-10-1819-s019.pdf]

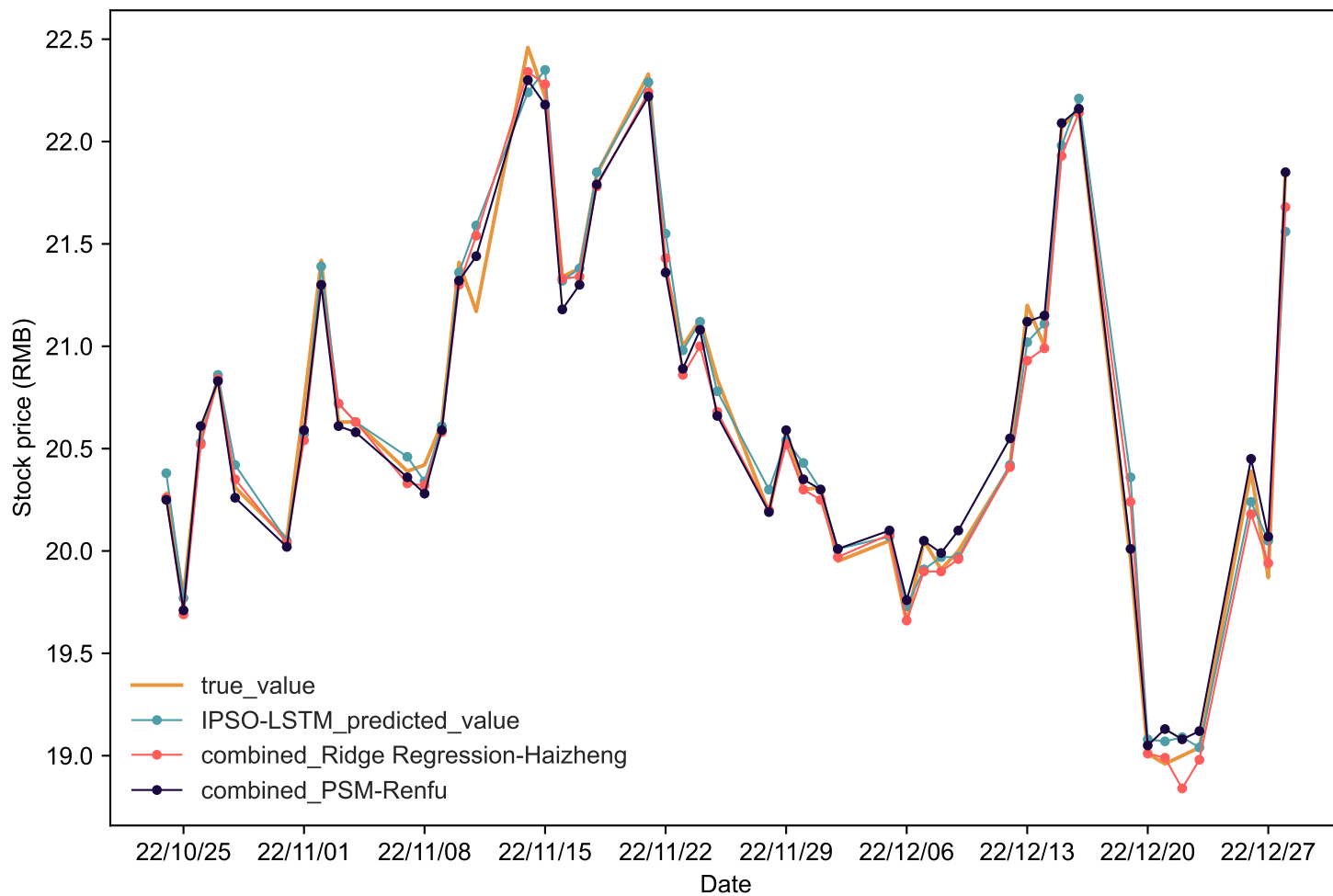

Supplement: Supplemental Information 20 — Note: True value is depicted by the orange line, the IPSO-LSTM model predictions without stock interdependencice are represented by the blue line, the red line indicates the IPSO-LSTM predictions considering ridge regression, and the IPSO-LSTM predictions considering PSM are illustrated by the black line. [file peerj-cs-10-1819-s020.pdf]

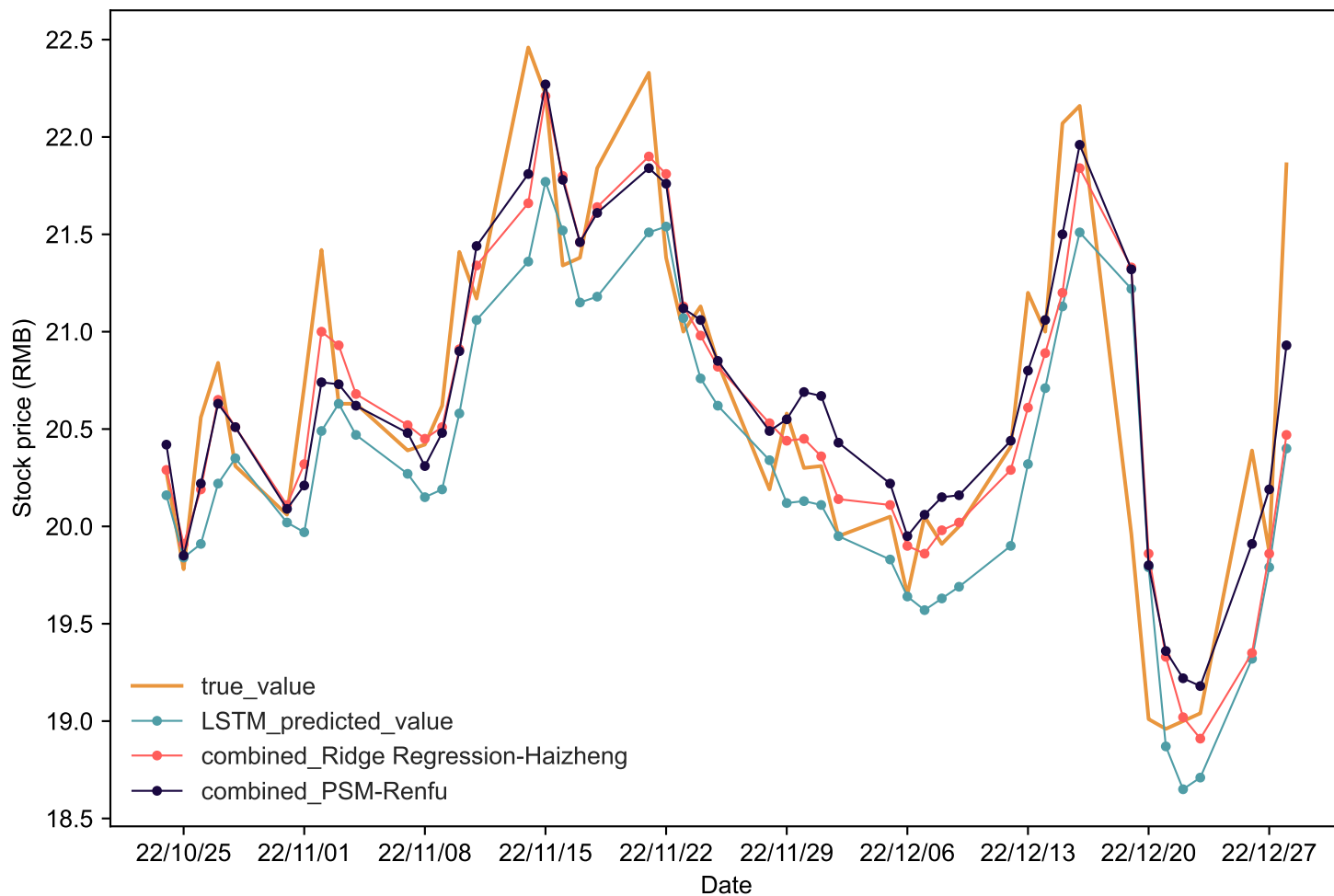

Supplement: Supplemental Information 21 — Note: True value is depicted by the orange line, the LSTM model predictions without stock interdependencice are represented by the blue line, the red line indicates the LSTM predictions considering ridge regression, and the LSTM predictions considering PSM are illustrated by the black line. [file peerj-cs-10-1819-s021.pdf]
