# Supplementary material for: The application of the propensity score matching method in stock prediction among stocks within the same industry
Source: PeerJ Comput Sci. 2024 Jan 30;10:e1819. doi: 10.7717/peerj-cs.1819 (PMC10909155; doi:10.7717/peerj-cs.1819)
Supplement: Supplemental Information 22 [file peerj-cs-10-1819-s022.docx]

**Table S1.** Results of balanced hypothesis testing for eight stock data pairs in the Biopharmaceuticals subsector.

| **Stocks** | **Variable** | **Unmatched** |  | **Mean** | |  |  | **%reduct** | |  | **T-test** | |  | **V(T)/** |
| --- | --- | --- | --- | --- | --- | --- | --- | --- | --- | --- | --- | --- | --- | --- |
|  |  | **Matched** |  | **Treated** | **Control** |  | **%Bias** | | **Bias** |  | **T** | **P>\|T\|** |  | **V(C)** |
| Junshi-Shenzhou |  | U |  | 55.446 | 70.046 |  | -111.8 | |  |  | -12.24 | 0.000 |  | 0.40^*^ |
|  | open | M |  | 56.618 | 56.097 |  | 4.0 | | 96.4 |  | 0.58 | 0.561 |  | 1.23 |
|  |  | U |  | 56.683 | 72.789 |  | -115.0 | |  |  | -12.60 | 0.000 |  | 0.38^*^ |
|  | high | M |  | 57.923 | 57.568 |  | 2.5 | | 97.8 |  | 0.37 | 0.712 |  | 1.12 |
|  |  | U |  | 54.232 | 67.817 |  | -112.3 | |  |  | -12.30 | 0.000 |  | 0.41^*^ |
|  | low | M |  | 55.367 | 54.709 |  | 5.4 | | 95.2 |  | 0.79 | 0.432 |  | 1.20 |
| Junshi-Baike |  | U |  | 57.43 | 70.046 |  | -97.7 | |  |  | -10.71 | 0.000 |  | 0.37^*^ |
|  | open | M |  | 59.844 | 60.316 |  | -3.7 | | 96.3 |  | -0.67 | 0.502 |  | 1.31 |
|  |  | U |  | 58.96 | 72.789 |  | -100.8 | |  |  | -11.04 | 0.000 |  | 0.32^*^ |
|  | high | M |  | 61.474 | 61.849 |  | -2.7 | | 97.3 |  | -0.53 | 0.599 |  | 1.18 |
|  |  | U |  | 55.938 | 67.817 |  | -97.9 | |  |  | -10.73 | 0.000 |  | 0.42^*^ |
|  | low | M |  | 58.288 | 58.971 |  | -5.6 | | 94.2 |  | -1.00 | 0.320 |  | 1.41^*^ |
| Junshi-Chengda |  | U |  | 50.43 | 70.046 |  | -139.7 | |  |  | -15.30 | 0.000 |  | 0.62^*^ |
|  | open | M |  | 55.36 | 54.137 |  | 8.7 | | 93.8 |  | 1.17 | 0.243 |  | 3.82^*^ |
|  |  | U |  | 50.947 | 72.798 |  | -147.9 | |  |  | -16.20 | 0.000 |  | 0.53^*^ |
|  | high | M |  | 55.995 | 54.703 |  | 8.7 | | 94.1 |  | 1.24 | 0.216 |  | 3.67^*^ |
|  |  | U |  | 49.901 | 67.817 |  | -134.4 | |  |  | -14.72 | 0.000 |  | 0.71^*^ |
|  | low | M |  | 54.684 | 53.366 |  | 9.9 | | 92.6 |  | 1.28 | 0.203 |  | 4.26^*^ |
| Junshi-Jindike |  | U |  | 47.672 | 70.046 |  | -172.4 | |  |  | -18.88 | 0.000 |  | 0.38^*^ |
|  | open | M |  | 55.29 | 55.578 |  | -2.2 | | 98.7 |  | -0.24 | 0.809 |  | 1.23 |
|  |  | U |  | 48.994 | 72.789 |  | -172.9 | |  |  | -18.94 | 0.000 |  | 0.33^*^ |
|  | high | M |  | 56.895 | 56.843 |  | 0.4 | | 99.8 |  | 0.04 | 0.966 |  | 1.01 |
|  |  | U |  | 46.454 | 67.817 |  | -175.6 | |  |  | -19.24 | 0.000 |  | 0.43^*^ |
|  | low | M |  | 53.858 | 54.303 |  | -3.7 | | 97.9 |  | -0.39 | 0.699 |  | 1.29 |
| Tiantan-Baiaotai |  | U |  | 21.725 | 23.303 |  | -59.5 | |  |  | -6.52 | 0.000 |  | 0.62^*^ |
|  | open | M |  | 21.761 | 22.004 |  | -9.2 | | 84.6 |  | -1.25 | 0.210 |  | 1.41^*^ |
|  |  | U |  | 22.123 | 23.707 |  | -59.3 | |  |  | -6.50 | 0.000 |  | 0.61^*^ |
|  | high | M |  | 22.149 | 22.413 |  | -9.9 | | 83.3 |  | -1.35 | 0.178 |  | 1.41^*^ |
|  |  | U |  | 21.319 | 22.936 |  | -62.4 | |  |  | -6.84 | 0.000 |  | 0.60^*^ |
|  | low | M |  | 21.355 | 21.537 |  | -7.0 | | 88.7 |  | -0.95 | 0.342 |  | 1.31^*^ |
| Jianyou-Kaiyin |  | U |  | 19.293 | 24.694 |  | -94.2 | |  |  | -10.32 | 0.000 |  | 0.18^*^ |
|  | open | M |  | 19.261 | 19.563 |  | -5.3 | | 94.4 |  | -0.95 | 0.343 |  | 0.74^*^ |
|  |  | U |  | 19.725 | 25.155 |  | -92.6 | |  |  | -10.15 | 0.000 |  | 0.20^*^ |
|  | high | M |  | 19.661 | 20.076 |  | -7.1 | | 92.4 |  | -1.21 | 0.226 |  | 0.69^*^ |
|  |  | U |  | 18.87 | 24.13 |  | -95.3 | |  |  | -10.44 | 0.000 |  | 0.17^*^ |
|  | low | M |  | 18.862 | 19.153 |  | -5.3 | | 94.5 |  | -0.97 | 0.333 |  | 0.72^*^ |
| Jianyou-Shansheng |  | U |  | 15.394 | 24.694 |  | -171.2 | |  |  | -18.76 | 0.000 |  | 0.06^*^ |
|  | open | M |  | 16.611 | 16.587 |  | 0.4 | | 99.7 |  | 0.13 | 0.896 |  | 1.92^*^ |
|  |  | U |  | 15.592 | 25.155 |  | -173.6 | |  |  | -19.02 | 0.000 |  | 0.06^*^ |
|  | high | M |  | 16.847 | 16.81 |  | 0.7 | | 99.6 |  | 0.21 | 0.838 |  | 1.65^*^ |
|  |  | U |  | 15.175 | 24.13 |  | -170.0 | |  |  | -18.62 | 0.000 |  | 0.07^*^ |
|  | low | M |  | 16.342 | 16.296 |  | 0.9 | | 99.5 |  | 0.26 | 0.799 |  | 1.92^*^ |
| Jianyou-Oulin |  | U |  | 20.126 | 24.694 |  | -69.4 | |  |  | -7.61 | 0.000 |  | 0.56^*^ |
|  | open | M |  | 20.084 | 20.329 |  | -3.7 | | 94.7 |  | -0.47 | 0.635 |  | 1.00 |
|  |  | U |  | 20.576 | 25.155 |  | -68.4 | |  |  | -7.49 | 0.000 |  | 0.57^*^ |
|  | high | M |  | 20.542 | 20.755 |  | -3.2 | | 95.4 |  | -0.41 | 0.685 |  | 1.01 |
|  |  | U |  | 19.563 | 24.13 |  | -72.1 | |  |  | -7.90 | 0.000 |  | 0.54^*^ |
|  | low | M |  | 19.555 | 19.748 |  | -3.0 | | 95.8 |  | -0.39 | 0.694 |  | 1.01 |
