# Supplementary material for: The application of the propensity score matching method in stock prediction among stocks within the same industry
Source: PeerJ Comput Sci. 2024 Jan 30;10:e1819. doi: 10.7717/peerj-cs.1819 (PMC10909155; doi:10.7717/peerj-cs.1819)
Supplement: Supplemental Information 23 [file peerj-cs-10-1819-s023.docx]

**Table S2.** Results of the common support test for eight pairs of stock data for the Biopharmaceuticals subsector.

| **Stocks** | **psmatch2: Treatment assignment** | **psmatch2: Common support** | | **Total** |
| --- | --- | --- | --- | --- |
|  |  | **Off support** | **On support** |  |
| Junshi-Shenzhou | Untreated | 18 | 222 | 240 |
|  | Treated | 25 | 215 | 240 |
|  | Total | 43 | 437 | 480 |
| Junshi-Baike | Untreated | 81 | 159 | 240 |
|  | Treated | 32 | 208 | 240 |
|  | Total | 113 | 367 | 480 |
| Junshi-Chengda | Untreated | 135 | 105 | 240 |
|  | Treated | 84 | 156 | 240 |
|  | Total | 219 | 261 | 480 |
| Junshi-Jindike | Untreated | 73 | 167 | 240 |
|  | Treated | 130 | 110 | 240 |
|  | Total | 203 | 277 | 480 |
| Tiantan-Baiaotai | Untreated | 41 | 199 | 240 |
|  | Treated | 2 | 238 | 240 |
|  | Total | 43 | 437 | 480 |
| Jianyou-Kaiyin | Untreated | 87 | 153 | 240 |
|  | Treated | 4 | 236 | 240 |
|  | Total | 91 | 389 | 480 |
| Jianyou-Shansheng | Untreated | 126 | 114 | 240 |
|  | Treated | 111 | 129 | 240 |
|  | Total | 237 | 243 | 480 |
| Jianyou-Oulin | Untreated | 12 | 228 | 240 |
|  | Treated | 4 | 236 | 240 |
|  | Total | 16 | 464 | 480 |
