# Supplementary material for: The application of the propensity score matching method in stock prediction among stocks within the same industry
Source: PeerJ Comput Sci. 2024 Jan 30;10:e1819. doi: 10.7717/peerj-cs.1819 (PMC10909155; doi:10.7717/peerj-cs.1819)
Supplement: Supplemental Information 24 — Note: ATT, Average Treatment Effect on the Treated; PSM, Propensity Score Matching. [file peerj-cs-10-1819-s024.docx]

**Table S3.** Data portfolios of stocks in the Biopharmaceuticals subsector with significant ATT and passing the PSM test.

| **Stocks** | **ATT** | **Mean propensity score** |
| --- | --- | --- |
| Junshi-Shenzhou | 6.90 | 0.50 |
| Junshi-Baike | 4.91 | 0.55 |
| Junshi-Chengda | 7.74 | 0.61 |
| Junshi-Jindike | 9.00 | 0.43 |
| Tiantan-Baiaotai | 4.30 | 0.53 |
| Jianyou-Kaiyin | 6.29 | 0.58 |
| Jianyou-Shansheng | 19.71 | 0.54 |
| Jianyou-Oulin | 5.06 | 0.51 |

Note: ATT, Average Treatment Effect on the Treated; PSM, Propensity Score Matching.
