# Supplementary material for: The application of the propensity score matching method in stock prediction among stocks within the same industry
Source: PeerJ Comput Sci. 2024 Jan 30;10:e1819. doi: 10.7717/peerj-cs.1819 (PMC10909155; doi:10.7717/peerj-cs.1819)
Supplement: Supplemental Information 25 — Note: Root Mean Square Error, RMSE; Mean Absolute Error, MAE; Mean Absolute Percentage Error, MAPE; coefficient of determination, R2. [file peerj-cs-10-1819-s025.docx]

**Table S4.** Evaluation of IPSO-LSTM model prediction results, comparing target stock independent prediction with matching stock prediction.

| **Stocks** | **Prediction Type** | **MAPE** | **RMSE** | **MAE** | **R^2^** |
| --- | --- | --- | --- | --- | --- |
| Junshi-Shenzhou | **independent** | 0.0191 | 1.3051 | 1.2323 | 0.9550 |
|  | **matching** | 0.0112 | 0.9358 | 0.7236 | 0.9769 |
| Junshi-Baike | **independent** | 0.0191 | 1.3051 | 1.2323 | 0.9550 |
|  | **matching** | 0.0097 | 0.7967 | 0.6285 | 0.9832 |
| Junshi-Chengda | **independent** | 0.0191 | 1.3051 | 1.2323 | 0.9550 |
|  | **matching** | 0.0078 | 0.7012 | 0.4941 | 0.9870 |
| Junshi-Jindike | **independent** | 0.0191 | 1.3051 | 1.2323 | 0.9550 |
|  | **matching** | 0.0058 | 0.4884 | 0.3683 | 0.9937 |
| Tiantan-Baiaotai | **independent** | 0.0057 | 0.1545 | 0.1260 | 0.9748 |
|  | **matching** | 0.0047 | 0.1310 | 0.1054 | 0.9819 |
| Jianyou-Kaiyin | **independent** | 0.0091 | 0.2039 | 0.1576 | 0.9010 |
|  | **matching** | 0.0074 | 0.1544 | 0.1277 | 0.9432 |
| Jianyou-Shansheng | **independent** | 0.0091 | 0.2039 | 0.1576 | 0.9010 |
|  | **matching** | 0.0066 | 0.1371 | 0.1134 | 0.9552 |
| Jianyou-Oulin | **independent** | 0.0091 | 0.2039 | 0.1576 | 0.9010 |
|  | **matching** | 0.0056 | 0.1192 | 0.0973 | 0.9661 |

Note: Root Mean Square Error, RMSE; Mean Absolute Error, MAE; Mean Absolute Percentage Error, MAPE; coefficient of determination, R^2^.
