# Supplementary material for: The application of the propensity score matching method in stock prediction among stocks within the same industry
Source: PeerJ Comput Sci. 2024 Jan 30;10:e1819. doi: 10.7717/peerj-cs.1819 (PMC10909155; doi:10.7717/peerj-cs.1819)
Supplement: Supplemental Information 26 [file peerj-cs-10-1819-s026.docx]

**Table S5.** Results of balanced hypothesis testing for three stock data pairs in the Proprietary Chinese Medicine subsector.

| **Stocks** | **Variable** | **Unmatched** |  | **Mean** | |  |  | **%reduct** | |  | **T-test** | |  | **V(T)/** |
| --- | --- | --- | --- | --- | --- | --- | --- | --- | --- | --- | --- | --- | --- | --- |
|  |  | **Matched** |  | **Treated** | **Control** |  | **%Bias** | | **Bias** |  | **T** | **P>\|T\|** |  | **V(C)** |
| Tongrentang-Xizang |  | U |  | 38.897 | 46.128 |  | -183.6 | |  |  | -20.12 | 0.000 |  | 1.29^*^ |
|  | open | M |  | 40.126 | 40.08 |  | 1.2 | | 99.4 |  | 0.13 | 0.898 |  | 1.10 |
|  |  | U |  | 39.622 | 47.195 |  | -187.4 | |  |  | -20.52 | 0.000 |  | 1.30^*^ |
|  | high | M |  | 40.884 | 40.806 |  | 1.9 | | 99.0 |  | 0.20 | 0.840 |  | 0.99 |
|  |  | U |  | 38.234 | 45.305 |  | -183.9 | |  |  | -20.14 | 0.000 |  | 1.23 |
|  | low | M |  | 39.417 | 39.088 |  | 8.6 | | 95.3 |  | 0.89 | 0.372 |  | 0.93 |
| Jichuan-Mayinglong |  | U |  | 22.389 | 25.978 |  | -145.1 | |  |  | -15.89 | 0.000 |  | 0.30^*^ |
|  | open | M |  | 23.063 | 23.049 |  | 0.6 | | 99.6 |  | 0.09 | 0.930 |  | 0.92 |
|  |  | U |  | 22.789 | 26.632 |  | -146.5 | |  |  | -16.05 | 0.000 |  | 0.30^*^ |
|  | high | M |  | 23.489 | 23.453 |  | 1.4 | | 99.0 |  | 0.21 | 0.831 |  | 0.92 |
|  |  | U |  | 22.026 | 25.399 |  | -144.0 | |  |  | -15.78 | 0.000 |  | 0.31^*^ |
|  | low | M |  | 22.671 | 22.576 |  | 4.1 | | 97.2 |  | 0.60 | 0.552 |  | 0.76 |
| Jichuan-Darentang |  | U |  | 25.08 | 25.978 |  | -28.3 | |  |  | -3.10 | 0.002 |  | 1.15 |
|  | open | M |  | 25.365 | 25.334 |  | 1.0 | | 96.6 |  | 0.11 | 0.912 |  | 1.46^*^ |
|  |  | U |  | 25.608 | 26.632 |  | -30.5 | |  |  | -3.35 | 0.001 |  | 1.12 |
|  | high | M |  | 25.917 | 25.903 |  | 0.4 | | 98.6 |  | 0.05 | 0.961 |  | 1.35^*^ |
|  |  | U |  | 24.55 | 25.399 |  | -28.1 | |  |  | -3.08 | 0.002 |  | 1.18 |
|  | low | M |  | 24.824 | 24.821 |  | 0.1 | | 99.6 |  | 0.01 | 0.990 |  | 1.43^*^ |
