# Supplementary material for: The application of the propensity score matching method in stock prediction among stocks within the same industry
Source: PeerJ Comput Sci. 2024 Jan 30;10:e1819. doi: 10.7717/peerj-cs.1819 (PMC10909155; doi:10.7717/peerj-cs.1819)
Supplement: Supplemental Information 27 [file peerj-cs-10-1819-s027.docx]

**Table S6.** Results of the common support test for three pairs of stock data for the Proprietary Chinese Medicine subsector.

| **Stocks** | **psmatch2: Treatment assignment** | **psmatch2: Common support** | | **Total** |
| --- | --- | --- | --- | --- |
|  |  | **Off support** | **On support** |  |
| Tongrentang-Xizang | Untreated | 1 | 239 | 240 |
|  | Treated | 47 | 193 | 240 |
|  | Total | 48 | 432 | 480 |
| Jichuan-Mayinglong | Untreated | 36 | 204 | 240 |
|  | Treated | 67 | 173 | 240 |
|  | Total | 103 | 377 | 480 |
| Jichuan-Darentang | Untreated | 6 | 234 | 240 |
|  | Treated | 15 | 225 | 240 |
|  | Total | 21 | 459 | 480 |
