# Supplementary material for: The application of the propensity score matching method in stock prediction among stocks within the same industry
Source: PeerJ Comput Sci. 2024 Jan 30;10:e1819. doi: 10.7717/peerj-cs.1819 (PMC10909155; doi:10.7717/peerj-cs.1819)
Supplement: Supplemental Information 28 — Note: ATT, Average Treatment Effect on the Treated; PSM, Propensity Score Matching. [file peerj-cs-10-1819-s028.docx]

**Table S7.** Data portfolios of stocks in the Proprietary Chinese Medicine subsector with significant ATT and passing the PSM test.

| **Stocks** | **ATT** | **Mean propensity score** |
| --- | --- | --- |
| Tongrentang-Xizang | 4.17 | 0.45 |
| Jichuan-Mayinglong | 9.46 | 0.47 |
| Jichuan-Darentang | 2.21 | 0.50 |

Note: ATT, Average Treatment Effect on the Treated; PSM, Propensity Score Matching.
