# Supplementary material for: The application of the propensity score matching method in stock prediction among stocks within the same industry
Source: PeerJ Comput Sci. 2024 Jan 30;10:e1819. doi: 10.7717/peerj-cs.1819 (PMC10909155; doi:10.7717/peerj-cs.1819)
Supplement: Supplemental Information 29 — Note: Root Mean Square Error, RMSE; Mean Absolute Error, MAE; Mean Absolute Percentage Error, MAPE; coefficient of determination, R2. [file peerj-cs-10-1819-s029.docx]

**Table S8.** Evaluation of IPSO-LSTM model prediction results, comparing target stock independent prediction with matching stock prediction.

| **Stocks** | **Prediction Type** | **MAPE** | **RMSE** | **MAE** | **R^2^** |
| --- | --- | --- | --- | --- | --- |
| Tongrentang-Xizang | **independent** | 0.0031 | 0.2440 | 0.1532 | 0.9887 |
|  | **matching** | 0.0027 | 0.1962 | 0.1292 | 0.9927 |
| Jichuan-Mayinglong | **independent** | 0.0050 | 0.2104 | 0.1580 | 0.9897 |
|  | **matching** | 0.0054 | 0.2007 | 0.1667 | 0.9906 |
| Jichuan-Darentang | **independent** | 0.0050 | 0.2104 | 0.1580 | 0.9897 |
|  | **matching** | 0.0048 | 0.1961 | 0.1522 | 0.9910 |

Note: Root Mean Square Error, RMSE; Mean Absolute Error, MAE; Mean Absolute Percentage Error, MAPE; coefficient of determination, R^2^.
