# Supplementary material for: The application of the propensity score matching method in stock prediction among stocks within the same industry
Source: PeerJ Comput Sci. 2024 Jan 30;10:e1819. doi: 10.7717/peerj-cs.1819 (PMC10909155; doi:10.7717/peerj-cs.1819)
Supplement: Supplemental Information 30 — Note: Rdige_coef, ridge regression coefficient. [file peerj-cs-10-1819-s030.docx]

**Table S9.** Ridge regression results for the four target stocks.

| **Stocks** | **Rdige_coef** |
| --- | --- |
| Hengrui-Zhangjiang | 0.36 |
| Fuxing-Yaoming | 0.30 |
| Fuxing-Borui | 0.33 |
| Renfu-Hengrui | 0.47 |
| Huahai-Haizheng | 0.54 |

Note: Rdige_coef, ridge regression coefficient.
