# Supplementary material for: The application of the propensity score matching method in stock prediction among stocks within the same industry
Source: PeerJ Comput Sci. 2024 Jan 30;10:e1819. doi: 10.7717/peerj-cs.1819 (PMC10909155; doi:10.7717/peerj-cs.1819)
Supplement: Supplemental Information 31 — Note: Root Mean Square Error, RMSE; Mean Absolute Error, MAE; Mean Absolute Percentage Error, MAPE; coefficient of determination, R2. [file peerj-cs-10-1819-s031.docx]

**Table S10.** Evaluation of prediction results of IPSO-LSTM and LSTM models, comparing PSM and ridge regression.

| **Prediction Models** | **Stocks** | **MAPE** | **RMSE** | **MAE** | **R^2^** |
| --- | --- | --- | --- | --- | --- |
| IPSO-LSTM | Hengrui_independent | 0.0059 | 0.2584 | 0.2388 | 0.9705 |
|  | Hengrui-Zhangjiang | 0.0044 | 0.2217 | 0.1748 | 0.9783 |
|  | Hengrui-Huana | 0.0034 | 0.1799 | 0.1332 | 0.9857 |
| LSTM | Hengrui _independent | 0.0154 | 0.7216 | 0.6029 | 0.7700 |
|  | Hengrui-Zhangjiang | 0.0136 | 0.6421 | 0.5360 | 0.8178 |
|  | Hengrui-Huana | 0.0123 | 0.6116 | 0.4874 | 0.8347 |

Note: Root Mean Square Error, RMSE; Mean Absolute Error, MAE; Mean Absolute Percentage Error, MAPE; coefficient of determination, R^2^.
