# Supplementary material for: The application of the propensity score matching method in stock prediction among stocks within the same industry
Source: PeerJ Comput Sci. 2024 Jan 30;10:e1819. doi: 10.7717/peerj-cs.1819 (PMC10909155; doi:10.7717/peerj-cs.1819)
Supplement: Supplemental Information 32 — Note: Root Mean Square Error, RMSE; Mean Absolute Error, MAE; Mean Absolute Percentage Error, MAPE; coefficient of determination, R2. [file peerj-cs-10-1819-s032.docx]

**Table S11.** Evaluation of prediction results of IPSO-LSTM and LSTM models, comparing PSM and ridge regression.

| **Prediction Models** | **Stocks** | **MAPE** | **RMSE** | **MAE** | **R^2^** |
| --- | --- | --- | --- | --- | --- |
| IPSO-LSTM | Fuxing_independent | 0.0086 | 0.3729 | 0.3072 | 0.9630 |
|  | Fuxing-Borui | 0.0075 | 0.3136 | 0.2674 | 0.9738 |
|  | Fuxing-Huana | 0.0040 | 0.2036 | 0.1466 | 0.9890 |
| LSTM | Fuxing_independent | 0.0227 | 1.0215 | 0.8194 | 0.7224 |
|  | Fuxing-Borui | 0.0175 | 0.8334 | 0.6318 | 0.8152 |
|  | Fuxing-Huana | 0.0169 | 0.7615 | 0.6056 | 0.8457 |
| IPSO-LSTM | Fuxing_independent | 0.0086 | 0.3729 | 0.3072 | 0.9630 |
|  | Fuxing-Yaoming | 0.0065 | 0.2823 | 0.2351 | 0.9788 |
|  | Fuxing-Hengrui | 0.0050 | 0.2131 | 0.1808 | 0.9879 |
| LSTM | Fuxing_independent | 0.0227 | 1.0215 | 0.8194 | 0.7224 |
|  | Fuxing-Yaoming | 0.0181 | 0.8553 | 0.6523 | 0.8054 |
|  | Fuxing-Hengrui | 0.0154 | 0.6766 | 0.5490 | 0.8782 |

Note: Root Mean Square Error, RMSE; Mean Absolute Error, MAE; Mean Absolute Percentage Error, MAPE; coefficient of determination, R^2^.
