# Supplementary material for: The application of the propensity score matching method in stock prediction among stocks within the same industry
Source: PeerJ Comput Sci. 2024 Jan 30;10:e1819. doi: 10.7717/peerj-cs.1819 (PMC10909155; doi:10.7717/peerj-cs.1819)
Supplement: Supplemental Information 33 — Note: Root Mean Square Error, RMSE; Mean Absolute Error, MAE; Mean Absolute Percentage Error, MAPE; coefficient of determination, R2. [file peerj-cs-10-1819-s033.docx]

**Table S12.** Evaluation of prediction results of IPSO-LSTM and LSTM models, comparing PSM and ridge regression.

| **Prediction Models** | **Stocks** | **MAPE** | **RMSE** | **MAE** | **R^2^** |
| --- | --- | --- | --- | --- | --- |
| IPSO-LSTM | Renfu_independent | 0.0054 | 0.1363 | 0.1182 | 0.9897 |
|  | Renfu-Hengrui | 0.0046 | 0.1241 | 0.0984 | 0.9915 |
|  | Renfu-Chengdu | 0.0036 | 0.1020 | 0.0757 | 0.9942 |
| LSTM | Renfu_independent | 0.0218 | 0.5842 | 0.4772 | 0.8112 |
|  | Renfu-Hengrui | 0.0194 | 0.5334 | 0.4261 | 0.8426 |
|  | Renfu-Chengdu | 0.0167 | 0.4629 | 0.3644 | 0.8814 |

Note: Root Mean Square Error, RMSE; Mean Absolute Error, MAE; Mean Absolute Percentage Error, MAPE; coefficient of determination, R^2^.
