# Supplementary material for: The application of the propensity score matching method in stock prediction among stocks within the same industry
Source: PeerJ Comput Sci. 2024 Jan 30;10:e1819. doi: 10.7717/peerj-cs.1819 (PMC10909155; doi:10.7717/peerj-cs.1819)
Supplement: Supplemental Information 34 — Note: Root Mean Square Error, RMSE; Mean Absolute Error, MAE; Mean Absolute Percentage Error, MAPE; coefficient of determination, R2. [file peerj-cs-10-1819-s034.docx]

**Table S13.** Evaluation of prediction results of IPSO-LSTM and LSTM models, comparing PSM and ridge regression.

| **Prediction Models** | **Stocks** | **MAPE** | **RMSE** | **MAE** | **R^2^** |
| --- | --- | --- | --- | --- | --- |
| IPSO-LSTM | Huahai_independent | 0.0043 | 0.1292 | 0.0893 | 0.9782 |
|  | Huahai-Haizheng | 0.0039 | 0.1146 | 0.0817 | 0.9829 |
|  | Huahai-Renfu | 0.0037 | 0.0955 | 0.0754 | 0.9881 |
| LSTM | Huahai_independent | 0.0203 | 0.5590 | 0.4243 | 0.5922 |
|  | Huahai-Haizheng | 0.0144 | 0.4475 | 0.3003 | 0.7386 |
|  | Huahai-Renfu | 0.0144 | 0.3993 | 0.2976 | 0.7919 |

Note: Root Mean Square Error, RMSE; Mean Absolute Error, MAE; Mean Absolute Percentage Error, MAPE; coefficient of determination, R^2^.
